# Sex differences in linear bone measurements occur following puberty but do not influence femoral or tibial torsion

**DOI:** 10.1038/s41598-023-38783-6

**Published:** 2023-07-20

**Authors:** Laura Carman, Thor Besier, N. Susan Stott, Julie Choisne

**Affiliations:** 1 Auckland Bioengineering Institute, The University of Auckland, Auckland, New Zealand; 2 Department of Engineering Science, The University of Auckland, Auckland, New Zealand; 3 Department of Surgery, Faculty of Medical and Health Sciences, The University of Auckland, Auckland, New Zealand

**Keywords:** Bone development, Bone, Bone, Paediatric research

## Abstract

Torsional, angular, and linear measurements in a paediatric population are clinically important but not well defined and understood. Different methods of measurement and discrepancies between assessors leads to a lack of understanding of what should be defined as typical or atypical for the growing skeleton. From a large dataset of 333 paediatric CT scans, we extracted three-dimensional torsional, angular, and linear measurements from the pelvis, femur, and tibia/fibula. Sex differences in linear measurements were observed in bones of children aged 13+ (around puberty), but femoral and tibial torsion were similar between males and females. The rotational profile (femoral anteversion minus tibial torsion) tended to increase with growth. Epicondylar, condylar, and malleolar widths were smaller in females than males for the same bone length after the age of 13 years, which could explain why females may be more at risk for sport injuries during adolescence. This rich dataset can be used as an atlas for researchers and clinicians to understand typical development of critical rotational profiles and linear bone measurements in children.

## Introduction

Skeletal growth in children is characterised by progressive modelling of the skeleton in response to a complex interplay between biological and mechanical factors^1^. Over half the skeletal growth occurs within the first few years of life, with the child at 2 years of age being half their adult height^2^. Bones continue to increase in size with age until growth plate fusion occurs, which can be up to 20 years of age in the lower limb^3^. As the limbs of the typically developing (TD) child grow, there is a programmed modelling of the long bones with systematic changes in length, width, and version^3^. Atypical development, for example a child with a neuromuscular disease, can lead to delayed or altered skeletal modelling with consequent effects on hip stability, gait pattern, and limb torsional alignment^4^. Understanding morphological variation in the typically developed skeleton is important for clinicians who diagnose and treat musculoskeletal pathology.

Angular deformities (sagittal and frontal plane) and torsional deformities (transverse plane) are particularly relevant, as they are exacerbated by altered mechanical loading^4,5^ and often targeted for reconstructive surgeries^6–8^. Increased femoral anteversion, for example, is common in children who are diagnosed with hip dysplasia and cerebral palsy^9,10^, yet defining the normal range of femoral anteversion is an area of debate^3,11^. As is the case for defining tibial torsion^11^. The mechanical lateral distal femoral angle (mLDFA) and mechanical medial proximal tibial angle (mMPTA) are also important for understanding the alignment of the knee joint and assisting surgical planning^12^, yet there is little paediatric data available to illustrate how these angles change with age^13^. It is also unclear when sexual dimorphism occurs in the bones of the growing skeleton.

Sex differences exist in adult linear bone measurements^14^ due to males typically being taller than females, therefore, size normalisation is needed to compare between sex. In the adult pelvis, sexual dimorphism results in similar normalised size (volumetric) but different shape between males and females^15^. Sex differences in knee morphology is an area of interest with investigations into sex-specific total knee replacements^16^. When normalising by distal depth, males have been shown to have a larger knee width than females^14,16,17^. The morphology of the tibia has also been shown to be smaller in females relative to body size^18^.

Sex differences in common clinical bone measurements have been demonstrated in adult cohorts but have not been widely studied in a TD paediatric population. Moreover, to the best of our knowledge, no studies have looked at automatically deriving clinical bone measurements from 3D bone reconstruction in a large cohort of TD children. We collected 333 CT scans of TD children aged from 4 to 18 years from which we extracted bone surface geometries of the pelvis, femora, and tibiae/fibulae. We subsequently developed an automatic workflow to identify bone landmarks and compute clinical bone measurements in the pelvis, femora, and tibiae/fibulae. Knowledge of how these values change with growth can help to inform the clinical community when making decisions for both TD children and children with bone abnormalities.

Although angular and torsional deformities are important for pathology, there are limited data that describe the normal variation of these measurements in the growing skeleton. Furthermore, challenges exist in comparing these data, due to methodological variation across these studies. Clinical bone measurements are typically taken by external clinical examination or medical imaging such as X-ray, CT, or MRI (with CT images considered as the gold standard^11^). However, discrepancies between imaging and measurement modalities^19^, make comparisons challenging. Fitting 3D models to medical imaging data can alleviate these challenges^20,21^, providing a consistent and reproducible frame of reference for future comparison^22^.

Therefore, our research objectives were to: (1) develop a consistent method for bone measurement calculation from 3D bone geometry, and (2) analyse age and sex differences in bone measurements and understand at which age these differences occur.

## Methods

### Bone segmentation

Retrospective post-mortem CT scans of 333 children [137 F, age: 4–18 years (12 ± 5 Y), height: 96–192 cm (148 ± 24 cm), mass: 14–140 kg (49 ± 22 kg)] were obtained from the Victorian Institute of Forensic Medicine (VIFM, Melbourne, Australia) with ethics approval EC 22/2016 from the VIFM Ethics Committee. This study used retrospective data, which was collected by the VIFM for autopsy purposes between 2006 and 2019. Before autopsy, the VIFM obtained written consent from the individual’s legal guardian. All methods were performed in accordance with the relevant guidelines and regulations. The distribution of participants by sex, age, and height can be found in Table 1.

**Table 1:** Total number of cases in each age (left) and height (right) category, grouped by sex, note age was only provided to the authors in years.

Age (years)	Female	Male	
4	10	16	
5	4	25	
6	3	13	
7	4	4	
8	8	10	
9	10	10	
10	2	8	
11	6	8	
12	7	9	
13	9	11	
14	11	17	
15	16	16	
16	15	17	
17	16	16	
18	16	16	
Total	137	196	

Height (cm)	Group name	Female	Male
95–99	95	3	1
100–104	100	2	5
105–109	105	2	10
110–114	110	5	18
115–119	115	4	10
120–124	120	7	8
125–129	125	1	9
130–134	130	8	10
135–139	135	7	5
140–144	140	3	9
145–149	145	6	8
150–154	150	8	6
155–159	155	17	8
160–164	160	30	8
165–169	165	22	22
170–174	170	11	31
175–179	175	0	12
180–184	180	1	11
185–189	185	0	2
190–195	190	0	3
Total		137	196

### Bone measurements

The pelvis, femora, and tibiae/fibulae were segmented from the CT scans and meshed into a 3D surface as outlined in a previous study^20^. A template mesh for each bone was then fit to each segmented bone model, to achieve nodal correspondence between meshes. This was achieved by (1) non-rigidly registering the template model and (2) iteratively mesh fitting the template model to the segmented data using radial basis functions^20^. Bones were aligned to an International Society of Biomechanics coordinate system convention^23^ using automatic detection of bony landmarks calculated from each 3D mesh to ensure all bones were in the same orientation for measurement. Bone measurements were automatically computed using code created in python from the calculated 3D landmarks for each mesh, as described below. Bone measurements were taken from both the left and right femora/tibiae.

#### Angular and torsional measurements

**Figure 1:**
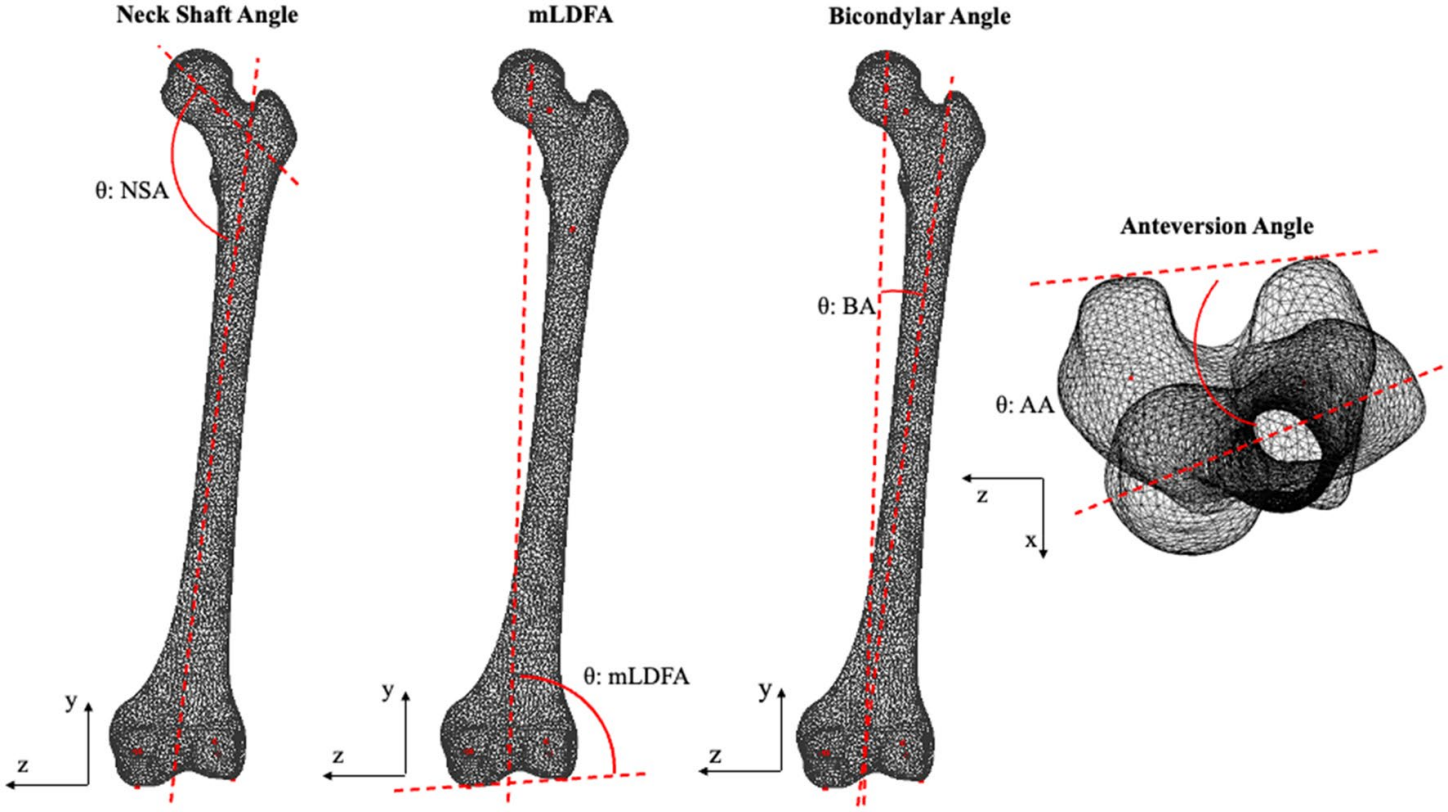
Angular measurements of the femur, where *NSA* neck shaft angle, *mLDFA* mechanical lateral distal femoral angle or femoral mechanical angle, *BA* bicondyar angle, *AA* anteversion angle.

**Figure 2:**
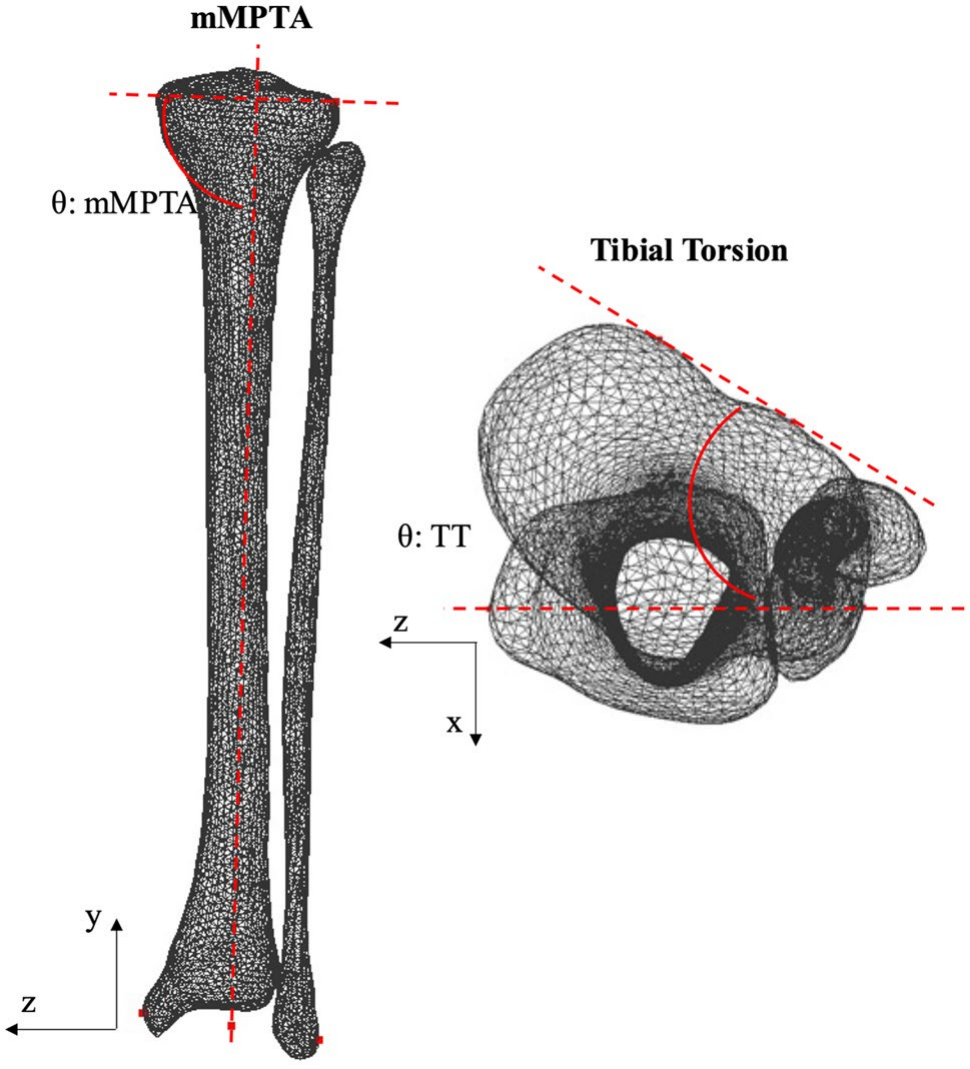
Angular measurements of the tibia/fibula, where *mMPTA* mechanical medial proximal tibial angle or tibial mechanical angle, *TT* tibial torsion.

- Anteversion angle: angle between the neck axis of the femur (measured between a sphere fit to the femoral head and the centre of a cylinder fitted to the femoral neck), and the posterior condylar axis (measured between the medial and lateral posterior femoral condyle) (Fig. 1)^12,24–32^.
- Neck shaft angle: angle between the neck axis of the femur and the shaft axis of the femur (measured between the centre of a cylinder fit to the femoral shaft below the lower trochanter and the midpoint of the centre of a cylinder fit to each femoral condyle) (Fig. 1)^33–35^.
- Femoral mechanical angle (mLDFA): angle between the knee axis of the femur (measured between the most distal points on the medial and lateral femoral condyles) and the vertical y-axis (measured between the femoral head centre and the condylar midpoint) (Fig. 1)^12,33,36–39^.
- Bicondylar angle: angle between a line perpendicular to the knee axis of the femur and the shaft axis of the femur (Fig. 1)^40–42^.
- Tibial torsion: angle between the posterior condylar axis (measured between the medial and lateral posterior tibial condyle) and the malleolar axis (measured between the medial and lateral malleolus) (Fig. 2)^24,26,29,30,43,44^.
- Tibial mechanical angle (mMPTA): angle between the knee axis of the tibia (measured between the centres of a cylinder fit to the medial and lateral tibial condyle) and the y-axis (measured between the midpoint of a cylinder fit to the medial and lateral tibial condyle and the midpoint between the medial and lateral malleolus) (Fig. 2)^12,33,36,38^.
- Rotational profile angle: tibial torsion minus anteversion angle.

Geometric fitting of a sphere to the femoral head was performed using the fitSphereAnalytic function found in the opensource python library GIAS3 (https://github.com/musculoskeletal/gias3). Geometric fitting of a cylinder to the femoral neck was performed using the python library cylinder_fitting (https://pypi.org/project/cylinder_fitting/#description). This was performed by manual selection of nodes from only the template mesh for the femoral head and neck to form a point cloud for geometric fitting. Fitting a sphere to the femoral head is a common method for accurate determination of the femoral head centre^28,45,46^. Cylinder fitting of the femoral neck is less common as often 3d geometry is not used for measurement, however this was determined to be the best approach from both manual inspection of the proximal femoral axis and existing literature^47,48^.

#### Linear measurements

**Figure 3:**
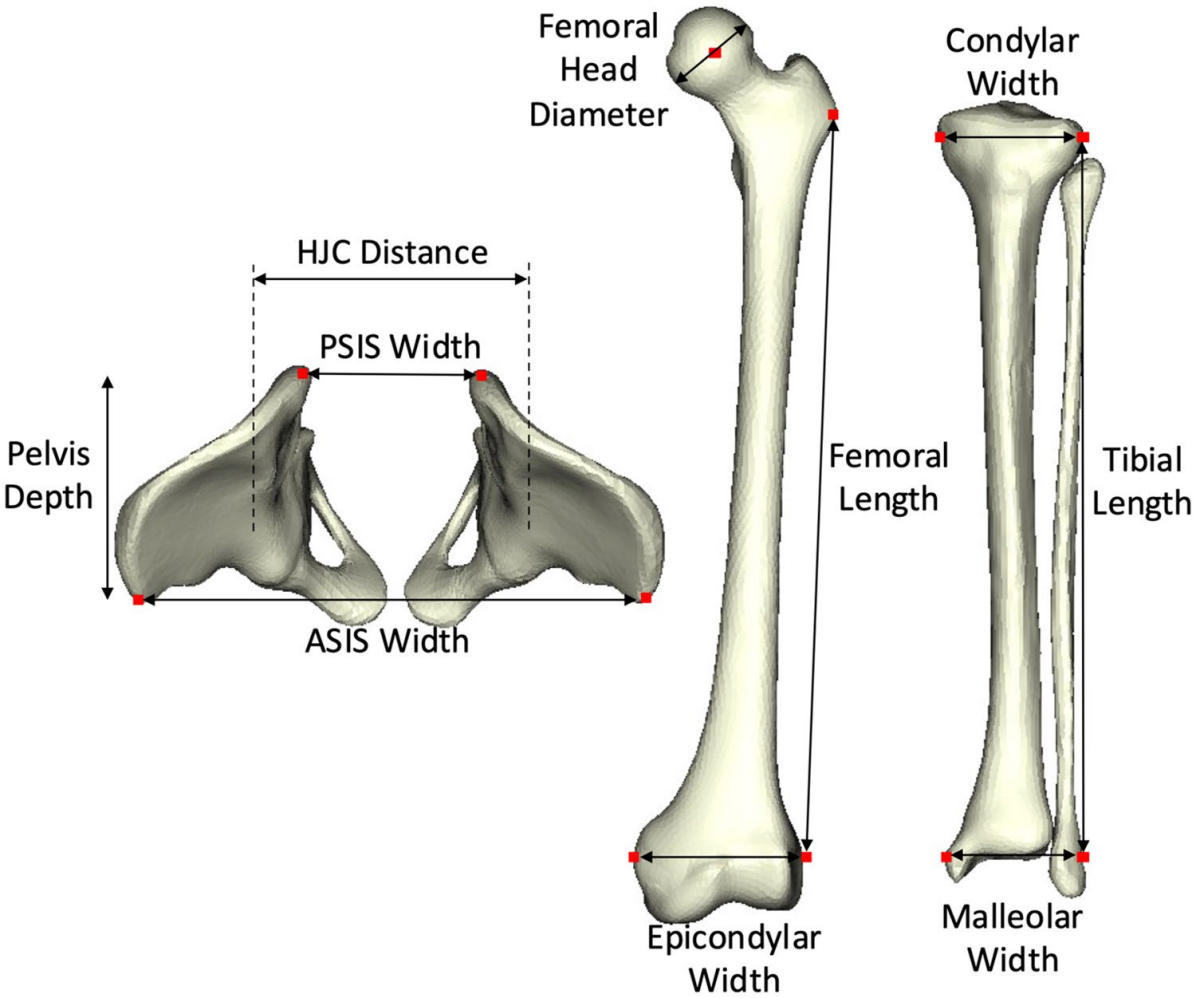
Linear bone measurements taken from the pelvis, femur, and tibia/fibula.

Computed linear measurements included Anterior Superior Iliac Spine (ASIS) width, Posterior Superior Iliac Spine (PSIS) width, pelvis depth, hip joint centre distance, femoral head diameter, femoral length, epicondylar width, condylar width, tibial length, and malleolar width (Fig. 3). Linear bone measurements were taken from automatically determined bony landmarks for each bone. The femoral length was determined by the distance between the greater trochanter and the lateral epicondyle bony landmarks. The tibial length was determined by the distance between the lateral condyle and the lateral malleolus of the tibia.

### Statistical analysis

Bone measurement results were grouped by sex and viewed in age and height groups. Age was recorded by the VIFM in years and height was measured manually using a tape measure for each participant. We also compared epicondylar width vs. femoral length, and condylar and malleolar width vs. tibial length. A linear regression analysis was performed to analyse the strength of the relationship between the response (bone measurements) and the explanatory variables (age/height). A two-way ANOVA was used to determine the influence of age/height and sex on each measurement and sex differences for each age/height category.

## Results

All measurement results and demographics are available to the public on the opensource platform SimTK.org (https://simtk.org/projects/paed_ssm). Sex differences in the results are only reported if the differences between groups reached a p value of < 0.05 when tested with a two-way ANOVA.

### Torsional measurements

**Figure 4:**
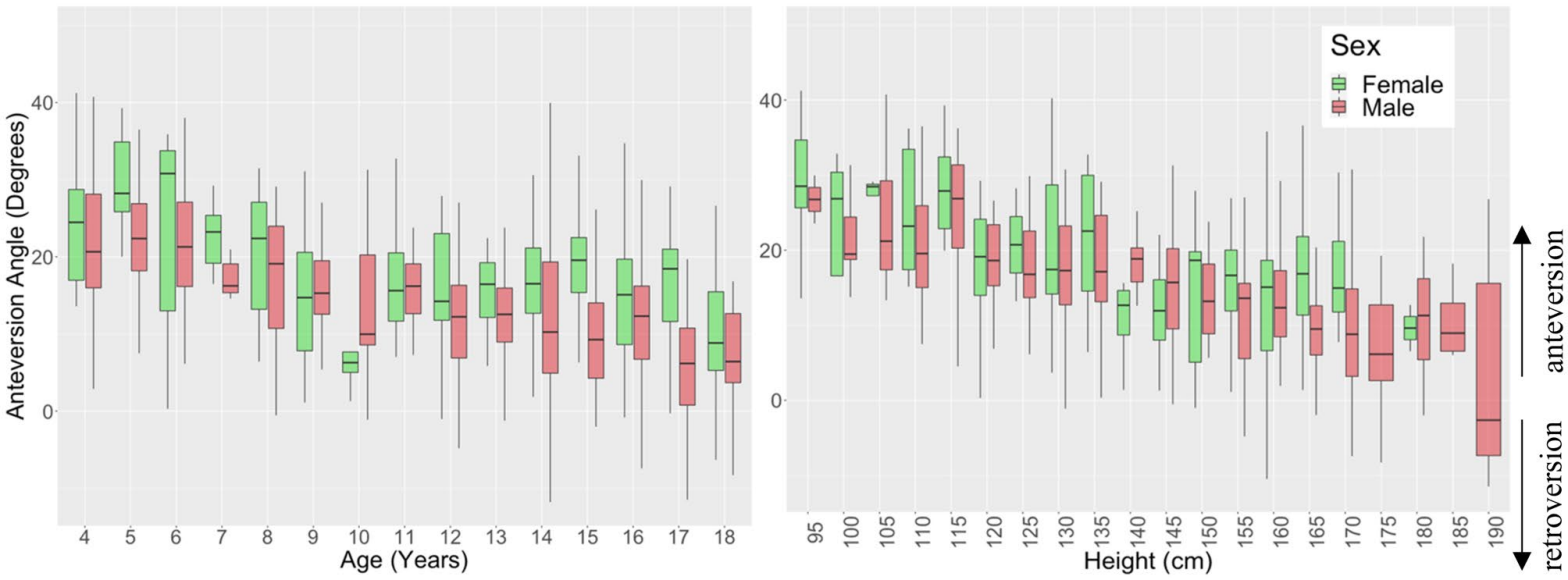
Anteversion angle vs. age (left) and height (right) for children aged 4–18 years grouped by sex.

**Figure 5:**
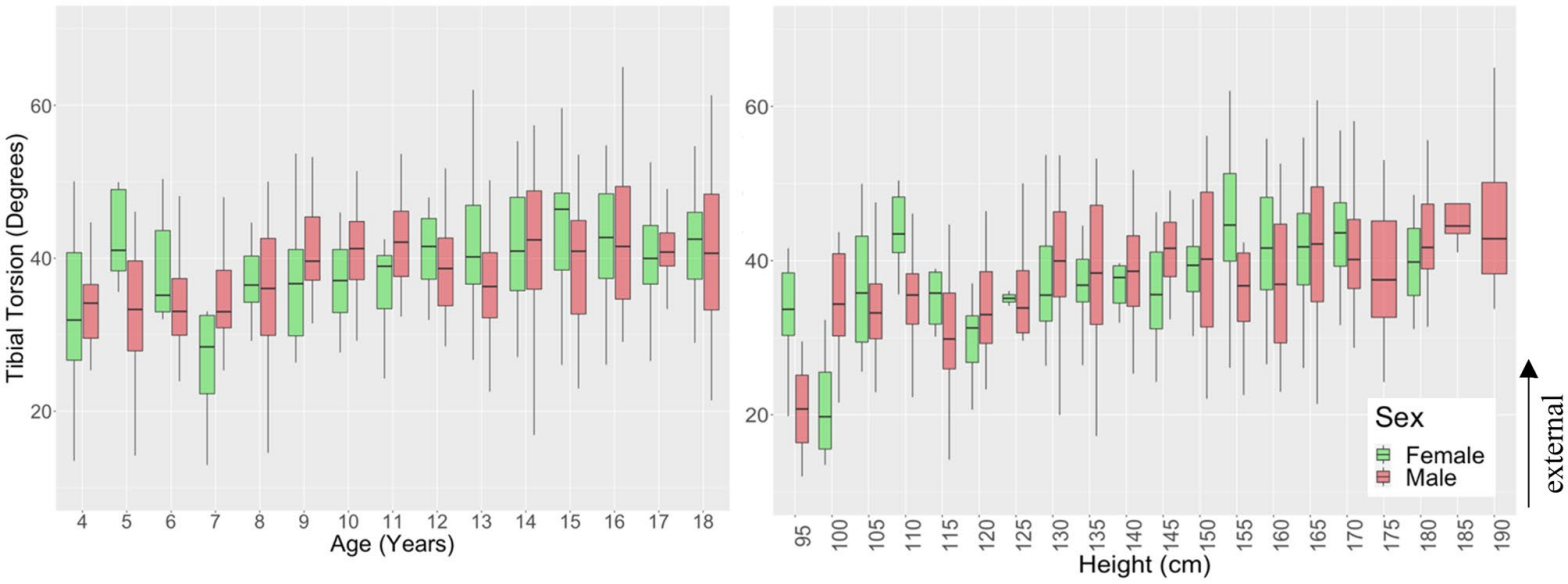
Tibial torsion vs. age (left) and height (right) for children aged 4–18 years grouped by sex.

Anteversion angle showed a weak decreasing relationship with age (R = − 0.4, p < 0.001) and height (R = − 0.42, p < 0.001) from an average of 22.0° ± 9.2° at 4 years of age to 8.8° ± 8.6° at 18 years (Fig. 4). Differences between males and females were found at 15 years and 17 years of age (p < 0.001) with females having a higher mean anteversion angle than males in these groups. Tibial torsion showed a weak increasing relationship with age (R = 0.36, p < 0.001) and height (R = 0.36, p < 0.001) from an average of 32.0° ± 10.1° at 4 years to 41.1° ± 8.5° at 18 years with sex differences between 155 and 159 cm in height (Fig. 5). The rotational profile angle (tibial torsion minus femoral anteversion angle) (Figure S6) showed an increasing relationship with age (R = 0.52, p < 0.001) and height (R = 0.54, p < 0.001) from an average of 9.8° ± 13.5° at 4 years to 32.3° ± 9.4° at 18 years.

### Angular measurements

**Figure 6:**
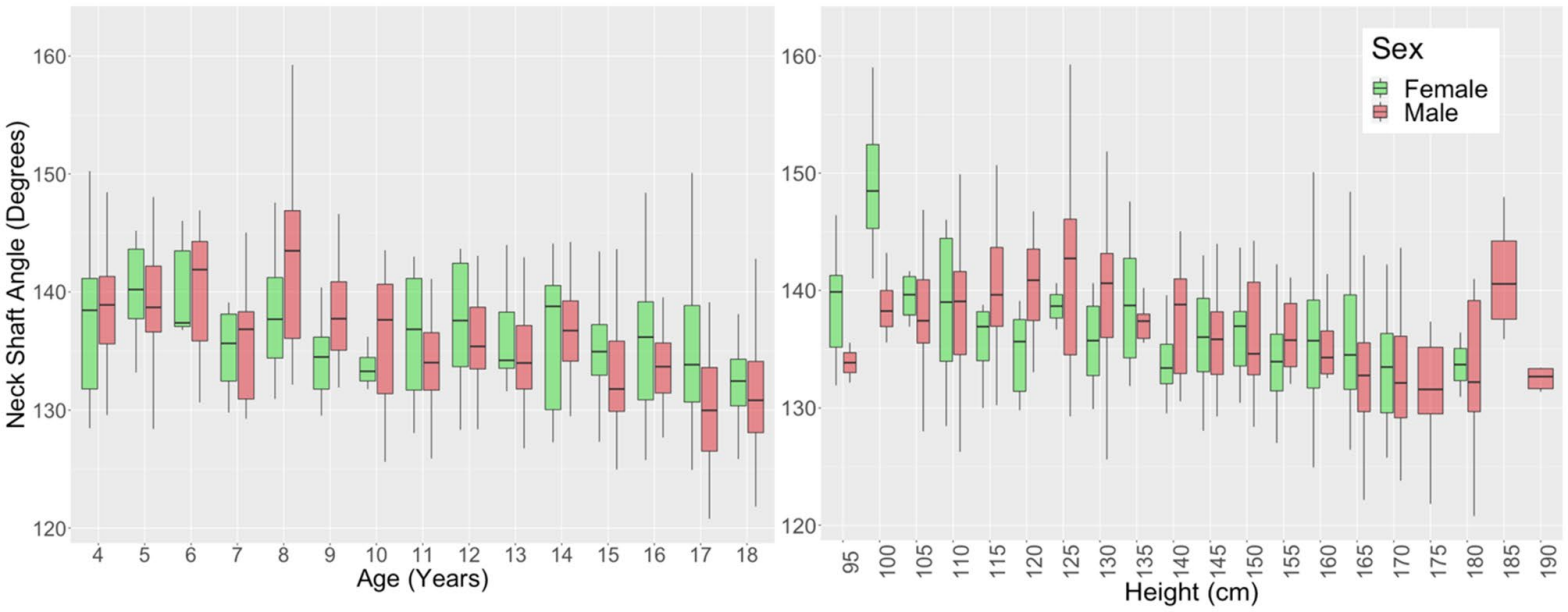
Neck shaft angle vs. age (left) and height (right) for children aged 4–18 years grouped by sex.

Neck shaft angle showed a weak decreasing relationship with age (R = − 0.39, p < 0.001) and height (R = − 0.37, p < 0.001) from an average of 138.6° ± 6.5° at 4 years to 132.1° ± 5.2° at 18 years (Fig. 6). Bicondylar angle increased from an average of 8.0° ± 2.2° to 9.7° ± 1.9° showing a weak relationship with age (R = 0.27, p < 0.001) and height (R = 0.31, p < 0.001) (Figure S1A,B). mLDFA showed a weak decreasing relationship with age (R = − 0.25, p < 0.001) and height (R = − 0.30, p < 0.001) (Figure S1C,D). mMPTA showed a weak decreasing relationship with age (R = − 0.30) and height (R = − 0.31) from an average of 90.1° ± 3.5° at 4 years to 87.8° ± 2.5° at 18 years (Figure S2A,B).

### Linear measurements

#### Pelvis

**Figure 7:**
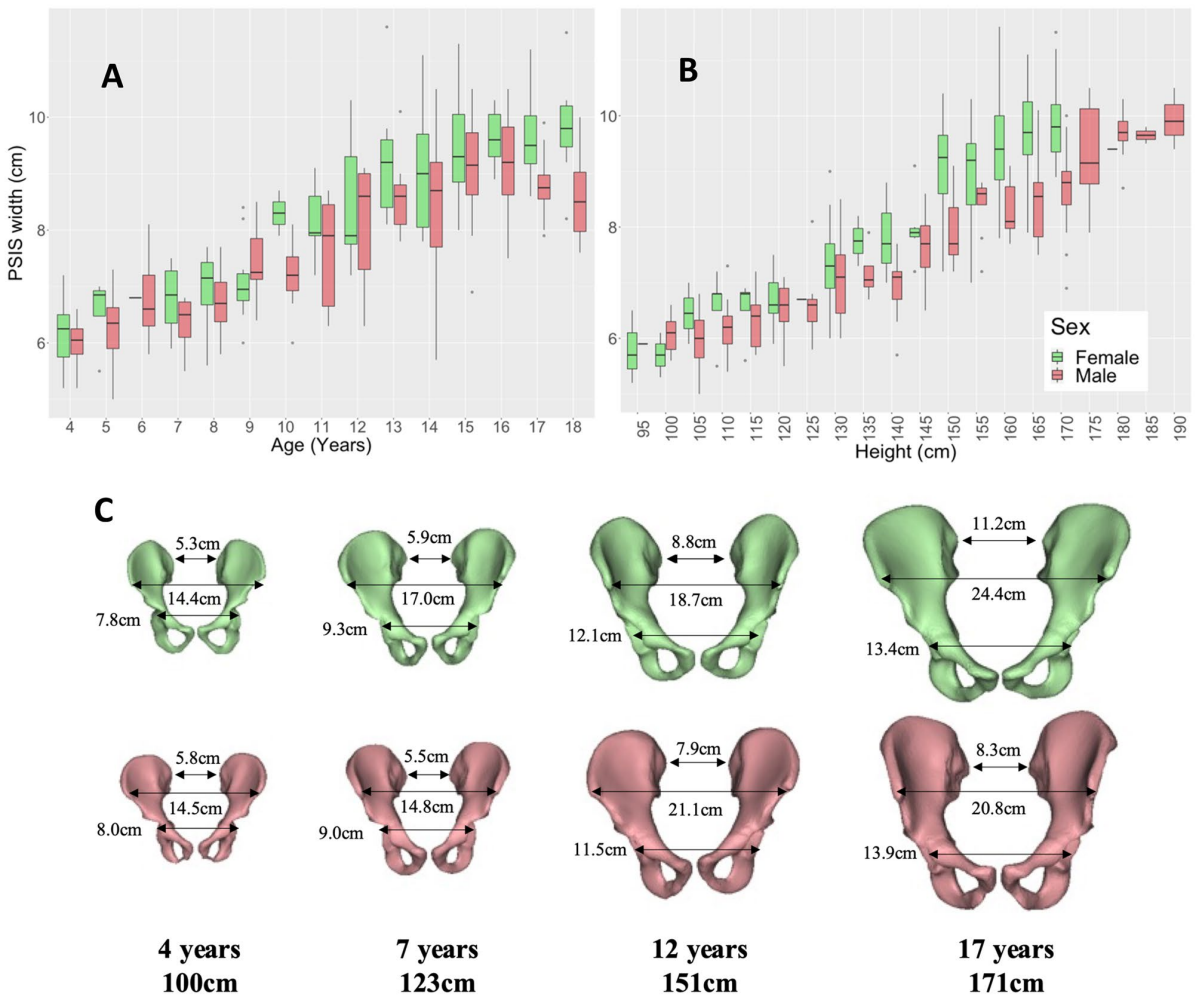
PSIS width vs. age (**A**) and height (**B**) for children aged 4–18 years grouped by sex. Example cases (**C**) show sex differences in ASIS width, PSIS width, and hip joint centre distance visually of height and aged matched cases.

ASIS width, PSIS width, pelvis depth, hip joint diameter and hip joint centre distance all showed strong increasing relationships with both age (R = 0.83, 0.82, 0.93, 0.90, and 0.93 respectively; p < 0.001) and height (R = 0.87, 0.83, 0.94, 0.98, and 0.93 respectively; p < 0.001) (Fig. 7C). ASIS width increased from an average of 14.6 ± 0.8 cm at 4 years to 21.8 ± 2.2 cm at 18 years (Figure S3A,B). PSIS width increased from an average of 6.1 ± 0.5 cm at 4 years to 9.2 ± 0.9 cm at 18 years with a distinction between males and females as age/height increases (Fig. 7A,B). Females had a larger PSIS width than males of the same age/height with differences from 18 years/160 cm (p < 0.05) (Table S1). Pelvis depth (Figure S3C,D) increased from an average of 7.5 ± 0.6 cm at 4 years to 14.2 ± 0.9 cm at 18 years with sex differences between the heights of 155–169 cm (p < 0.05) where females had a larger pelvis depth than males of the same height (Table S1). Hip joint diameter (Figure S3E,F) increased from an average of 4.2 ± 0.3 cm at 4 years to 6.6 ± 0.3 cm at 18 years with sex differences at 15 years (p = 0.03) where males had a larger hip joint diameter (Table S1). Hip joint centre distance increased from an average of 8.6 ± 0.5 cm at 4 years to 13.8 ± 0.7 cm at 18 years (Figure S3G,H) with differences between males and females between 160 and 164 cm (p < 0.05) with females having a larger hip joint centre distance than males in this height range (Table S1).

#### Femur

**Figure 8:**
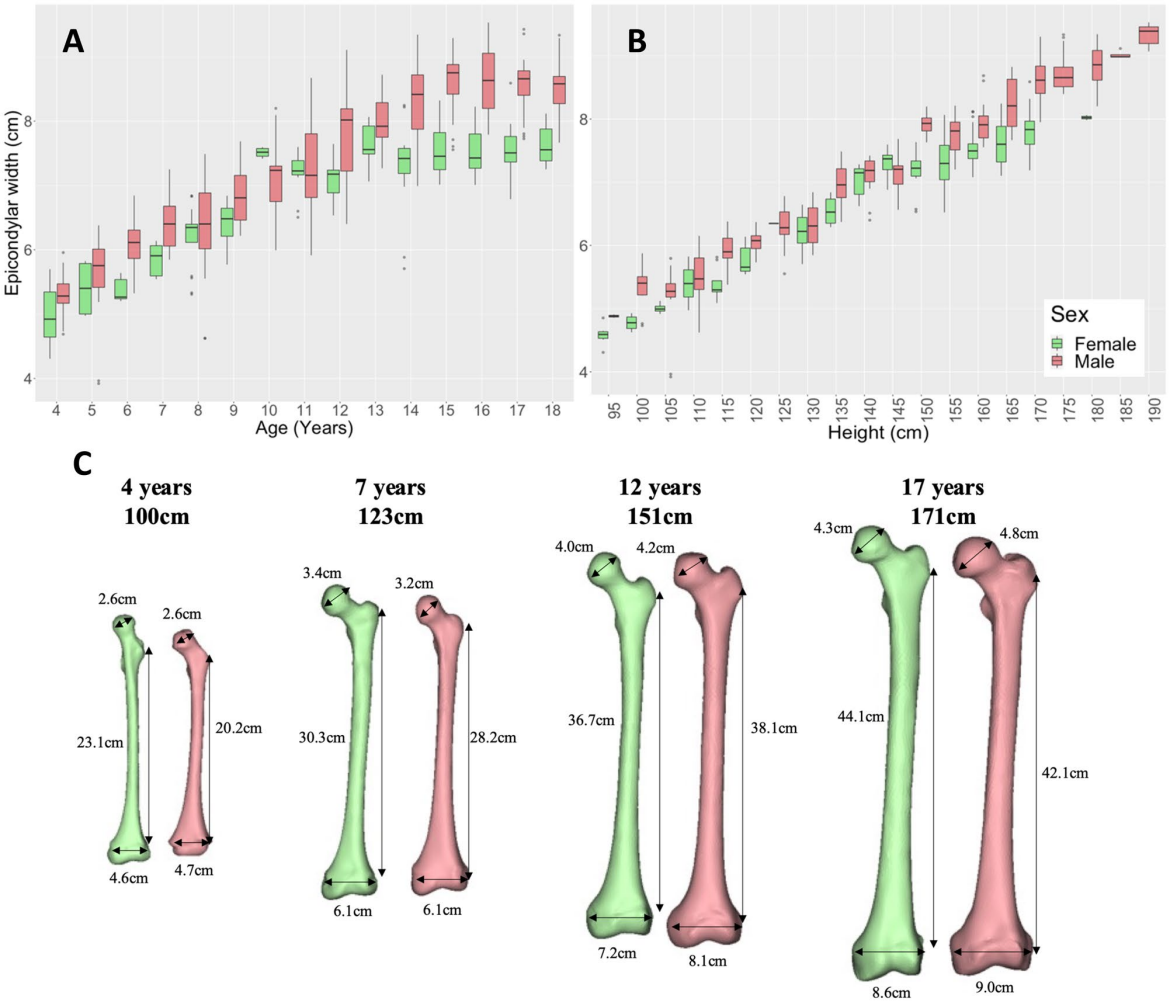
Epicondylar width vs. age (**A**) and height (**B**) for children aged 4–18 years grouped by sex. Example cases (**C**) show sex differences in femoral head diameter, femoral length, and epicondylar width visually of height and aged matched cases.

Femoral length, epicondylar width, and femoral head diameter showed an increasing relationship with age (R = 0.91, 0.84, and 0.89 respectively; p < 0.001) and height (R = 0.98, 0.94, and 0.96 respectively; p < 0.001) (Fig. 8C). Epicondylar width increased from an average of 5.2 ± 0.4 cm at 4 years to 8.1 ± 0.6 cm at 18 years (Fig. 8A,B). Femoral head diameter increased from an average of 2.7 ± 0.2 cm at 4 years to 4.5 ± 0.3 cm at 18 years (Figure S1E,F). Femoral length increased from an average of 22.6 ± 1.8 cm at 4 years to 40.3 ± 2.2 cm at 18 years (Figure S1G,H). These measurements all showed sex differences from age 14 onwards (p < 0.05) with males having larger linear measurements compared to females of the same age/height (Table S1). Interesting to note is the divergence in the epicondylar width of males and females after the age of 13. The epicondylar width in females remained static after age 13 but continued to increase in males up until the age of 15 (Fig. 8A). Epicondylar width showed sex differences between 115–119 cm and 150–174 cm in height (p < 0.05). When comparing epicondylar width to femoral length (Figure S4) sex differences were seen between femoral lengths 28–29 cm, and 38–45 cm (p < 0.05), with females having a smaller epicondylar width compared to males for the same femoral length.

#### Tibia/fibula

**Figure 9:**
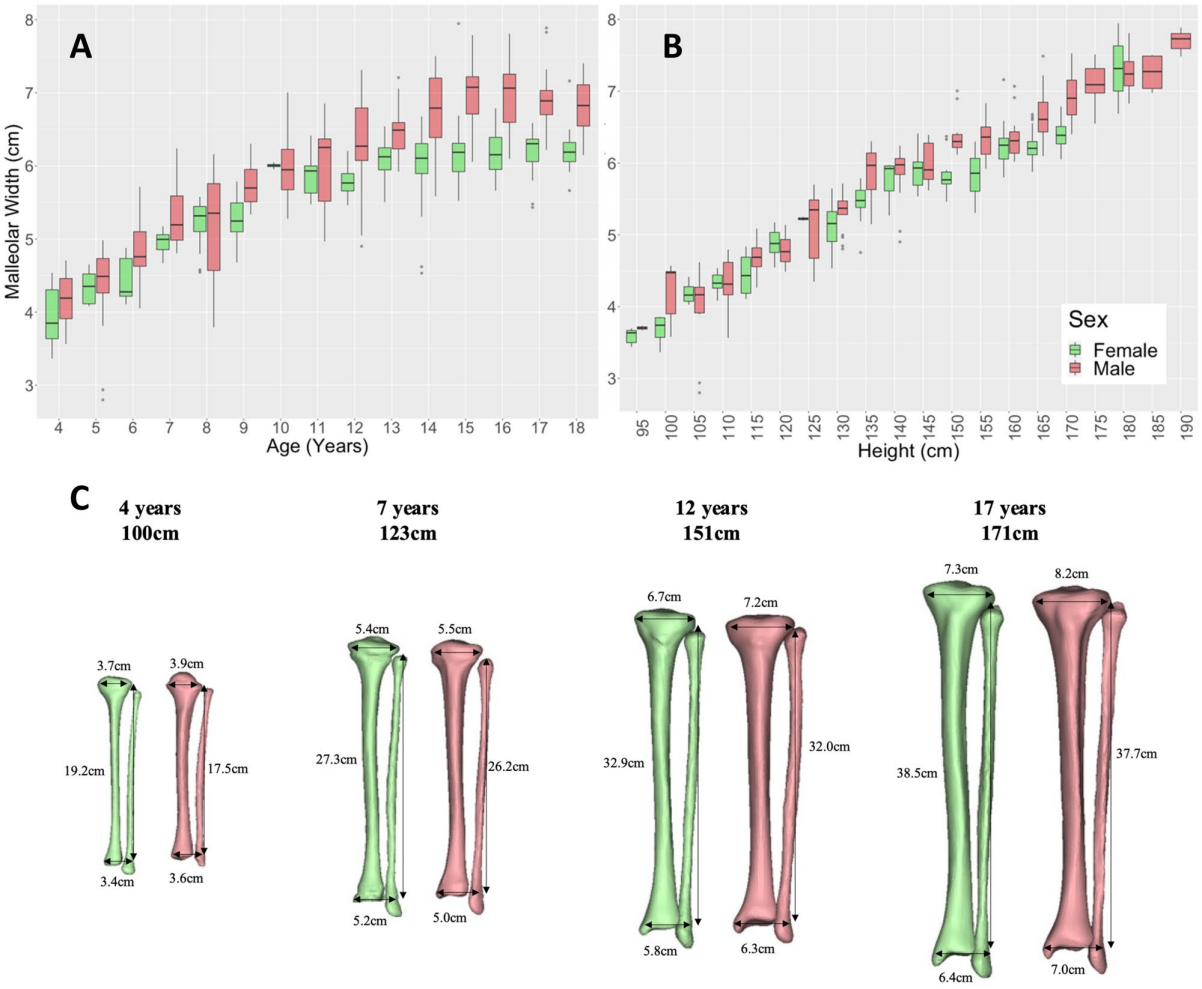
Malleolar width vs. age (**A**) and height (**B**) for children aged 4–18 years grouped by sex. Example cases (**C**) show sex differences in tibial length, condylar width, and malleolar width visually of height and aged matched cases.

Tibial length, condylar width, and malleolar width showed increasing relationships with age (R = 0.89, 0.87, and 0.83 respectively; p < 0.001) and height (R = 0.98, 0.96, and 0.94 respectively; p < 0.001) (Fig. 9C). Malleolar width increased from an average of 4.1 ± 0.4 cm at 4 years to 6.5 ± 0.4 cm at 18 years (Fig. 9A,B). Condylar width increased from an average of 4.2 ± 0.4 cm at 4 years to 7.5 ± 0.5 cm at 18 years (Figure S2C,D). Tibial length increased from an average of 20.1 ± 2.0 cm at 4 years to 36.2 ± 2.4 cm at 18 years (Figure S2E,F). Sex differences were observed in these measurements from age 14 onwards (p < 0.05) with males having larger linear measurements compared to females of the same age/height (Table S1). Condylar width showed sex differences from 165 cm in height (p < 0.05). Malleolar width showed sex differences between 150 and 159 cm in height from 165 cm (p < 0.05) (Table S1), with males having larger condylar and malleolar widths compared to females in these height groups. Similar to the femur, we found a distinction between males and females from around the age of 13; small change in condylar and malleolar width was seen in females but males exhibited increased joint width until 15 years old (Fig. 9A, Fig. S2C). When comparing condylar and malleolar width to tibial length (Figure S5) sex differences were seen from a tibial length of 36 cm between males and females (p < 0.05), with females having smaller joint widths in both cases for the same tibial length.

## Discussion

The objectives of our study were to: (1) develop a consistent method for bone measurement calculation from 3D bone geometry, and (2) analyse age and sex differences in bone measurements and understand at which age these differences occur. A method for automatically and reliably computing bone measurements for the pelvis, femora, and tibiae/fibulae was developed and applied to a dataset of 333 children aged 4–18 years. Clinical bone measurements were extracted across the population and statistical analysis was performed. Key findings were: (1) the torsional profile of the femur did not vary with sex, except at the ages 15 and 17 years; (2) the torsional profile of the tibia was not influenced by sex; (3) angular measurements of the femur (neck shaft angle, bicondylar angle and mLDFA) and tibia (mMPTA) did not reveal any sex differences with age or height; (4) sex differences were observed in the linear measurements of the pelvis (PSIS width and hip joint diameter), femora (femoral head diameter, epicondylar width and femoral length) and tibiae (condylar and malleolar width and tibial length).

### Torsional measures

Reported torsional bone measurements for the femur and tibia/fibula vary widely in the literature. This is likely due to different methods of data collection (clinical or radiographic) and measurement protocols^5,19,49^. For femoral anteversion, these measures include using clinical tests such as maximum lateral trochanteric prominence related to the degree of internal rotation of the hip in prone, X-rays, MRI, or CT^11,49^. For tibial torsion, equivalent measures are transmalleolar axis, CT, and MRI^50^. To the authors knowledge, there are no existing studies which have calculated torsional measures for both femur and tibia from 3D bone geometry in children.

In the current study, the femoral anteversion angle decreased with age from an average of 22° at 4 years to 9° at 18 years over the dataset. Sex differences were only found for femoral anteversion at ages 15 and 17 years, consistent with previous adult studies, showing that females have a larger anteversion angle by about 5°^12,25,29^. Greater change occurred in first 8–10 years of life, with smaller changes after that age continuing up to age 18 years. This decrease over time is also consistent with previous studies, with femoral anteversion for TD children reported to decrease on average from 40° at birth, to 24° at 10 years, 20° by mid to late adolescence and 16° in adulthood^5,10,30,51–55^. It is important to bear in mind that multiple methods to measure femoral anteversion exist. These result in 16°–20° differences across the full range of anteversion for the same participant resulting in reported anteversion angles for adults between 7° and 24°^5,49^. This study showed decreasing femoral anteversion with age, greatest in the first 8–10 years, but continuing into (and past) skeletal maturity. These findings support other studies which suggest that femoral anteversion may continue to slowly decrease into adulthood^5^, for reasons not well explained.

This study also showed a gradual increase in external tibial torsion with age from about 32° to 41°. This is consistent with existing studies which found increasing tibial torsion from 27° to 35° from 6 to 30 years of age^56^ and from 34° to 36° in a 3 to 10 year age group^57^. However, the range of reported tibial torsion varies greatly and only a few studies have investigated children. The measurements from the current study on 3-D CT were slightly more lateral (external) than the reported clinical measures. CT measures of tibial torsion define rotation between the proximal and distal ends of the tibia. However, clinical measures assess tibial rotation relative to a flexed knee position, which induces some medial rotation of the tibia^58^. As a result, CT measures of tibial torsion can be more lateral than clinical measures^50^ but likely reflect true bony torsion. Sex differences for tibial torsion were only observed between 155 and 159 cm in height, consistent with the adult literature stating no sex differences in the adult population^56^.

This study demonstrated the wide range of anteversion angle and tibial torsion that can be considered as normal in a paediatric population. Although sex differences are minimal, the anteversion and tibial torsion angles have been shown to moderately decrease and increase respectively with age with an overall increasing torsional profile during growth.

### Angular measurements

Angular measurements showed small variation with age and height and few sex differences. Neck shaft angle decreased with age from an average of about 139° to about 132°, which is consistent with previous studies where femoral neck shaft angle has been shown to generally decrease from childhood to adulthood^59^ (from ~ 150° at birth to ~ 140° aged nine years, to ~ 128° in adulthood^3,10,53,59,60^). mLDFA on average was between 84° and 87° in this study, which is slightly lower than a previous study which found mLDFA values of 87°–88° in children aged 4–18 years^13^ and adult values ranging from 87° to 91°^12,37,38^. However, few studies have reported this angle in a paediatric population. mMPTA on average was between 86° and 90°. This is consistent with a previous study which found mMPTA values of 88°–90° in children aged 4–18 years^13^ and adult values ranging from 82° to 88°^12,37,38^.

No sex differences were found for the neck shaft angle and mLDFA in this dataset, however, differences have been reported in adult studies^12,25,38,59^. For example, neck shaft angle was found to be higher and mLDFA to be lower in females by 1°–2°^12,25,38,59^. These sex differences may take longer to develop in the lower limb bones once the bone has stopped growing and is modelled based on loads experienced. mMPTA showed sex differences only at 17 years of age, which is supported by previous studies in adults with females showing 1°–2° higher mMPTA^37–39^.

Overall femoral and tibial angular measurements do not appear to be influenced by sex and age/height. Worth noting is the large standard deviation in any angular measurements for a given age/height, which highlights the variability of these measurements in a typically developed population.

### Linear measurements

Pelvic, femoral, and tibial linear measurements increased with age and height as was expected and sex differences were observed for many of the measurements. The sexual dimorphism in the pelvis is of interest in the field of obstetrics. In this study, the most marked sex difference was in the PSIS width with an increase in PSIS width in females at 18 years old and from 160 cm in height, which is consistent with the shape differences found between the adult female and male pelvis^15^ and the posterior space being more influenced by sex^61^. Sex differences were seen in the hip joint centre distance between 155 and 164 cm in height which is also consistent with literature^62^.

For all linear measurements of the femur and tibia/fibula, sex differences were seen beyond 13 years old and at various height ranges. When comparing to bone length, the epicondylar width showed sex differences from a femoral length of 38 cm onward, with females having smaller epicondylar width compared to males for the same femoral length. Similarly for the tibia/fibula, sex differences were observed from a tibial length of 36 cm onward with condylar and malleolar width being smaller in females compared to males for a similar bone length. This is consistent with prior literature comparing the adult knee between females and males^14,17,18^ and highlights the importance of patient-specific treatment protocols such as orthopaedic implant size selection and sports injury prevention.

### Limitations

This study has several limitations to be considered when interpreting the results. First, the dataset was obtained from a subset of the population living in the state of Victoria in Australia with 75% of the population living in an urbanised area which would be similar to most developed countries but could be different from groups living in more rural areas. While ethnicity was not recorded for this data, the top five ethnicities recorded in the state of Victoria are English (24%), Australian (22%), Irish (8%), Scottish (7%), and Chinese (5%) (Australian census, 2021). Both factors could potentially impact measurements and the applicability of these findings to other populations. Additionally, some age groups had fewer number of CT scans (Table 1) meaning the population in these age groups may not be fully represented. The sex distribution is also not equal for some of the age/height groups therefore caution is advised when interpreting sex differences in these groups. As the data was de-identified, we had access to participant age in years (not months) at the time of the CT scan. Therefore, a child referenced as 4 years old may be 4 years and 1 month old or 4 years and 11 months old, during which time large growth changes may occur. Therefore, we also examined these measurement changes with height. Also, inferences regarding morphological bone changes due to growth should be made with caution, as these data are cross-sectional, rather than longitudinal. Cadaveric stature was measured in this study rather than standing/living stature which may result in differences in participant height. When conducting the statistical analysis, both left and right bone measurements were used for the femora and tibiae/fibulae, we acknowledge that there is some variation in limb length for an individual and that it is less than the variation between individuals. This may affect the averages presented for each age/height grouping.

To use the automated bone measurement methods developed in this study, three-dimensional bone surfaces reconstructed from full lower limb CT or MRI scans are required. This limits the application of our method, however, we have also developed statistical shape models that can be used to predict the bone morphology from sparse anatomical landmarks^20^. Future integration of these methods will enable clinicians to compare patient-specific data to our population norms.

## Conclusions

We developed an automated workflow to compute lower limb bone measurements and rotational profiles in children aged 4–18 years. We found sex differences in the linear bone measurements in the pelvis, femur, and tibia/fibula beyond the age of 13 years. This rich dataset can be used as an atlas for researchers and clinicians to understand typical development of critical rotational profiles and linear bone measurements in children. Finally, all computed bone measurements and code for automatically extracting bone measurements from 3D point clouds is available on the opensource platform SimTK.org (https://simtk.org/projects/paed_ssm).

## Supplementary Information

Supplementary Information.

## Data Availability

All data generated or analysed during this study are included in this published article and its supplementary information files. Raw data is available to the public at the following link: https://simtk.org/projects/paed_ssm.
