# Supplementary material for: Sex differences in linear bone measurements occur following puberty but do not influence femoral or tibial torsion
Source: Sci Rep. 2023 Jul 20;13:11733. doi: 10.1038/s41598-023-38783-6 (PMC10359265; doi:10.1038/s41598-023-38783-6)
Supplement: Supplementary file 1 — Supplementary Information. [file 41598_2023_38783_MOESM1_ESM.docx]

**Supplementary Tables and Figures: Sex differences in linear bone measurements occur following puberty but do not influence femoral or tibial torsion**

Laura Carman^1^, Thor Besier^1, 2^, N Susan Stott^3^, Julie Choisne^1*^

^1^Auckland Bioengineering Institute, The University of Auckland, Auckland, New Zealand

^2^Department of Engineering Science, The University of Auckland, Auckland, New Zealand

^3^Department of Surgery, Faculty of Medical and Health Sciences, The University of Auckland, New Zealand

* [j.choisne@auckland.ac.nz](mailto:j.choisne@auckland.ac.nz)

Table S1: Significant gender differences in all clinical measurements taken. ‘Significant ages/heights’ gives the individual age/height groups at which there are significant differences between males and females, where significance is classified as p < 0.05. Factor significance gives the overall significance of each factor in the relationship, where ‘***’ p<0.001, ‘**’ p=0.001, ‘*’ p=0.01, ‘.’ P=0.05.

|  | **Measurement** | **Significant Ages (years)** | **Factor significance** | | | **Significant Heights (cm)** | **Factor Significance** | | |
| --- | --- | --- | --- | --- | --- | --- | --- | --- | --- |
|  |  |  | **Sex** | **Age** | **Age*Sex** |  | **Sex** | **Height** | **Height*Sex** |
| **Pelvis** | **ASIS width** | N/A | ** | *** |  | N/A | ** | *** |  |
|  | **PSIS width** | 18 | *** | *** |  | >160 | *** | *** | * |
|  | **Hip joint diameter** | 15 | * | *** | * | N/A | ** | *** |  |
|  | **Pelvis depth** | N/A | *** | *** |  | 155-169 | *** | *** | ** |
|  | **Hip joint centre distance** | N/A | *** | *** |  | 160-164 | *** | *** | *** |
| **Femur** | **Anteversion angle** | 15, 17 | *** | *** | * | N/A | *** | *** | . |
|  | **Neck shaft angle** | N/A |  | *** | *** | N/A |  | *** | ** |
|  | **Bicondylar angle** | N/A |  | *** | *** | N/A |  | *** | ** |
|  | **Mechanical lateral distal femoral angle (mLDFA)** | N/A | . | *** | *** | N/A | . | *** | ** |
|  | **Femoral head diameter** | >13 | *** | *** | *** | 115-119, 150-159, 165-174 | *** | *** | *** |
|  | **Epicondylar width** | >13 | *** | *** | *** | 115-119, 150-174 | *** | *** | *** |
|  | **Femoral length** | >13 | *** | *** | *** | N/A | *** | *** | *** |
| **Tibia/Fibula** | **Tibial torsion** | N/A | ** | *** | . | 155-159 | ** | *** | *** |
|  | **Mechanical medial proximal tibial angle (mMPTA)** | 17 | * | *** | * | N/A | * | *** | ** |
|  | **Condylar width** | >13 | *** | *** | *** | >165 | *** | *** | *** |
|  | **Malleolar width** | >13 | *** | *** | *** | 150-159, >165 | *** | *** | *** |
|  | **Tibial length** | >13 | ** | *** | *** | 160-164, 180-184 | *** | *** | *** |
|  | **Rotational Profile Angle** | N/A |  | *** |  | N/A |  | *** |  |

| \|  \| **Femur** \| \| \| --- \| --- \| --- \| \|  \| **Age** \| **Height** \| \| **Bicondylar Angle** \| 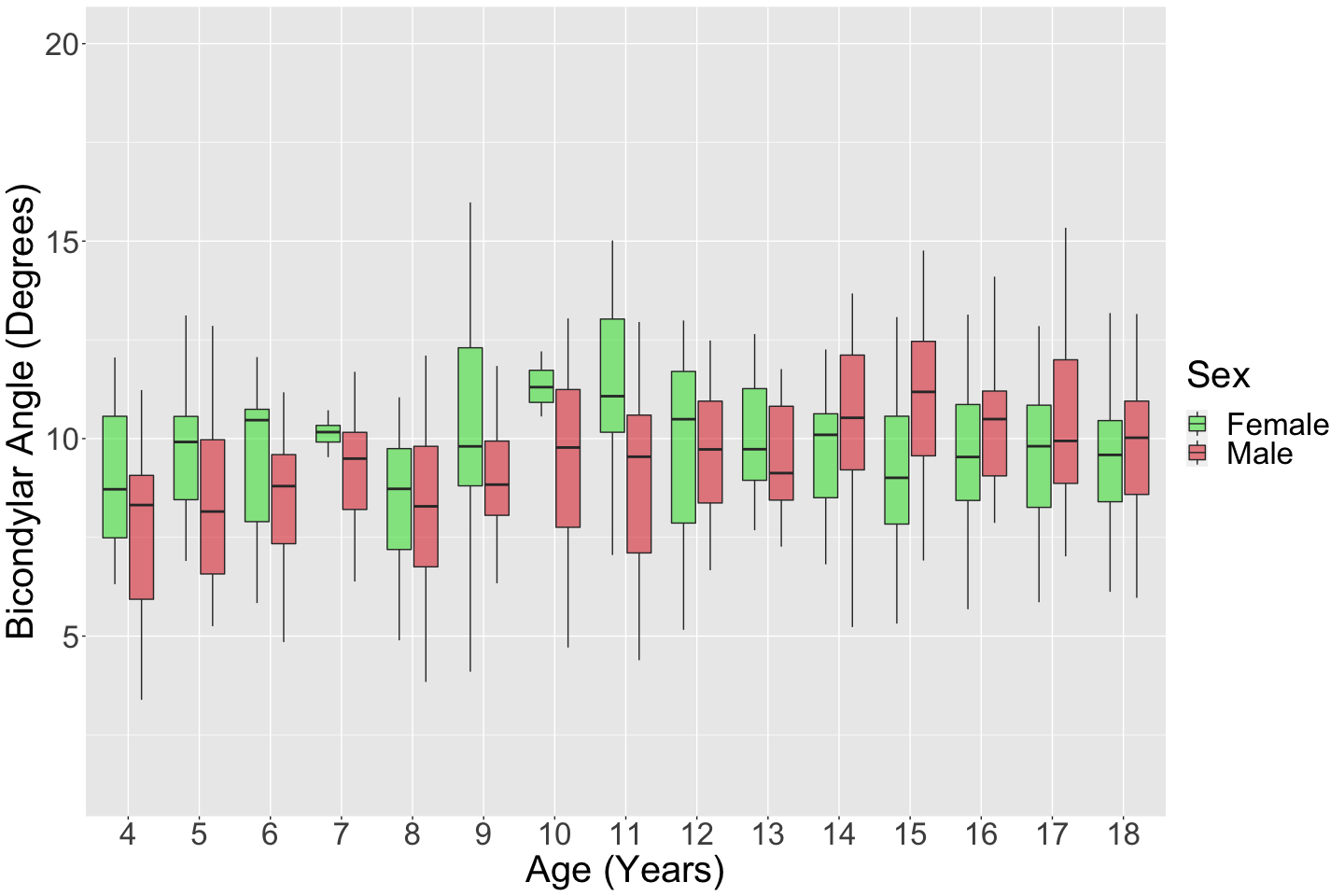  **A** \| 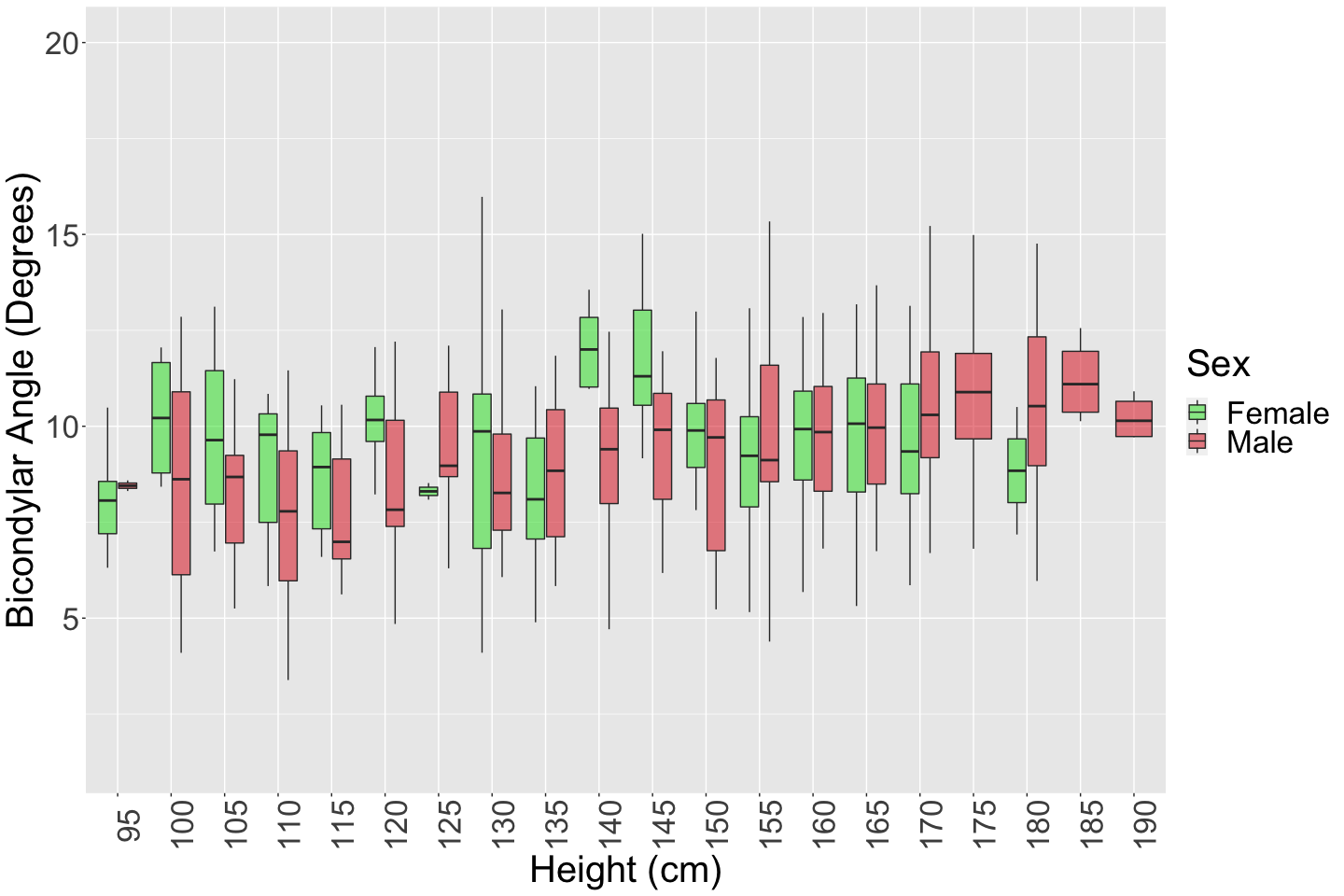  **B** \| \| **mLDFA** \| 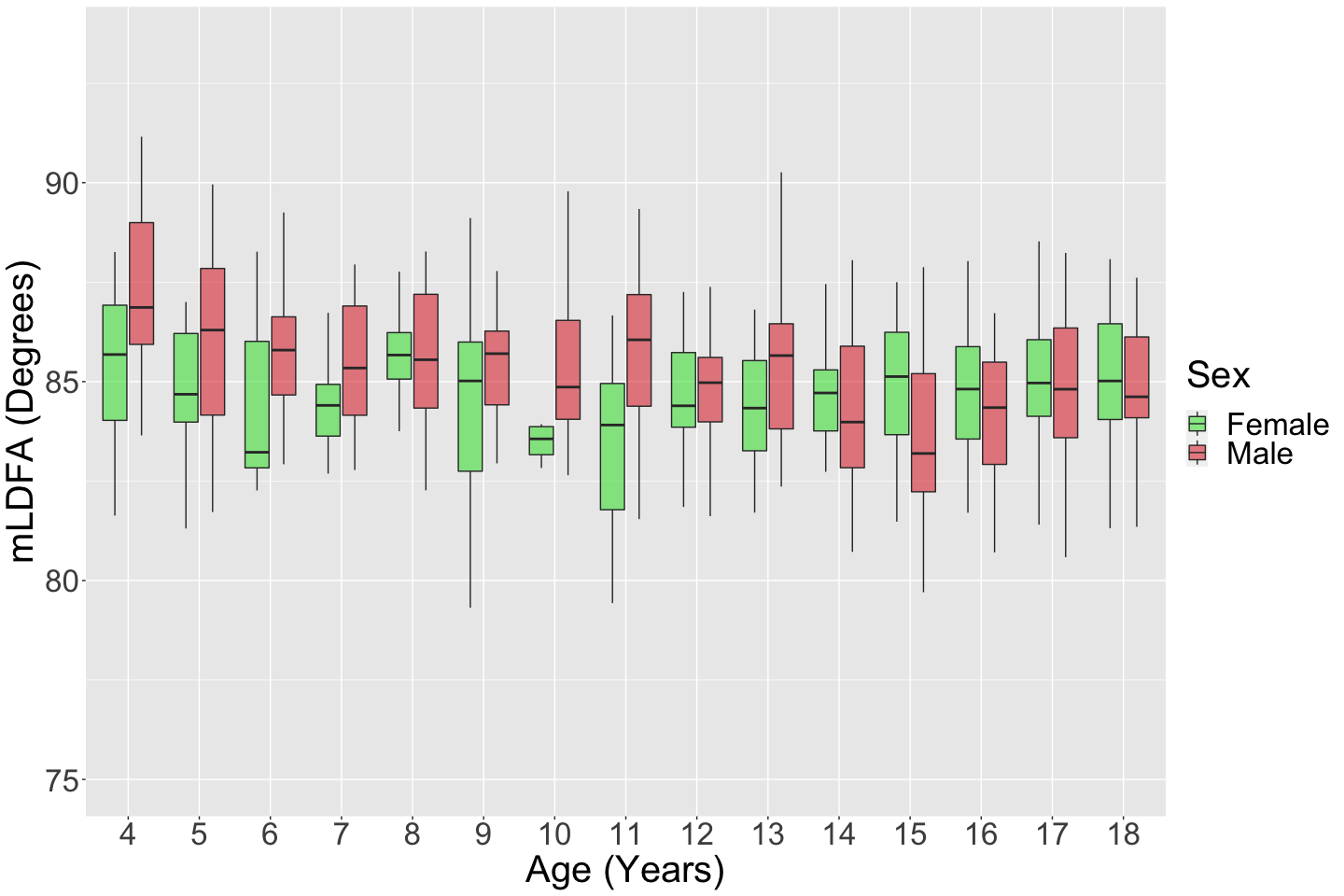  **C** \| 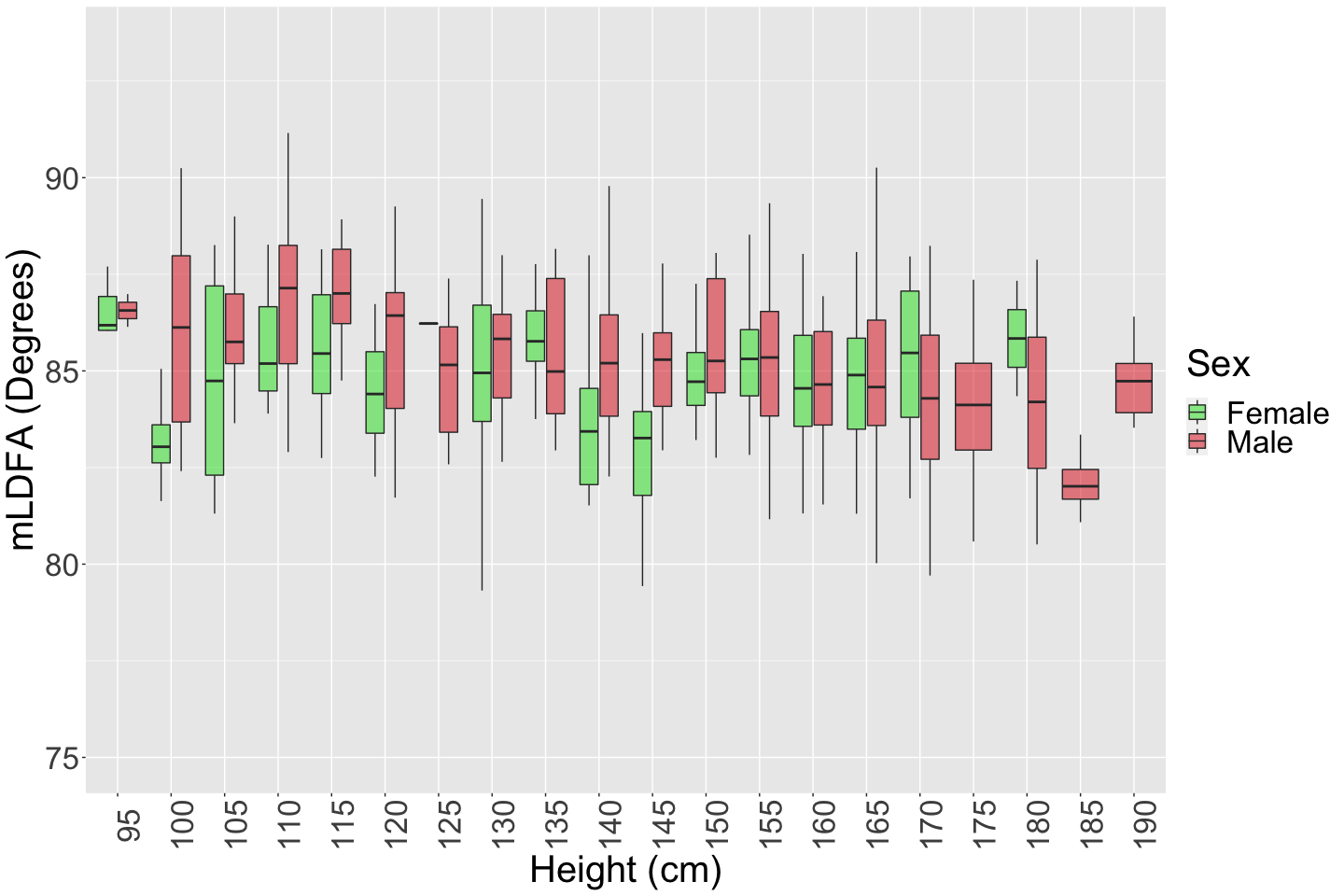  **D** \| \| **Femoral Head Diameter** \| 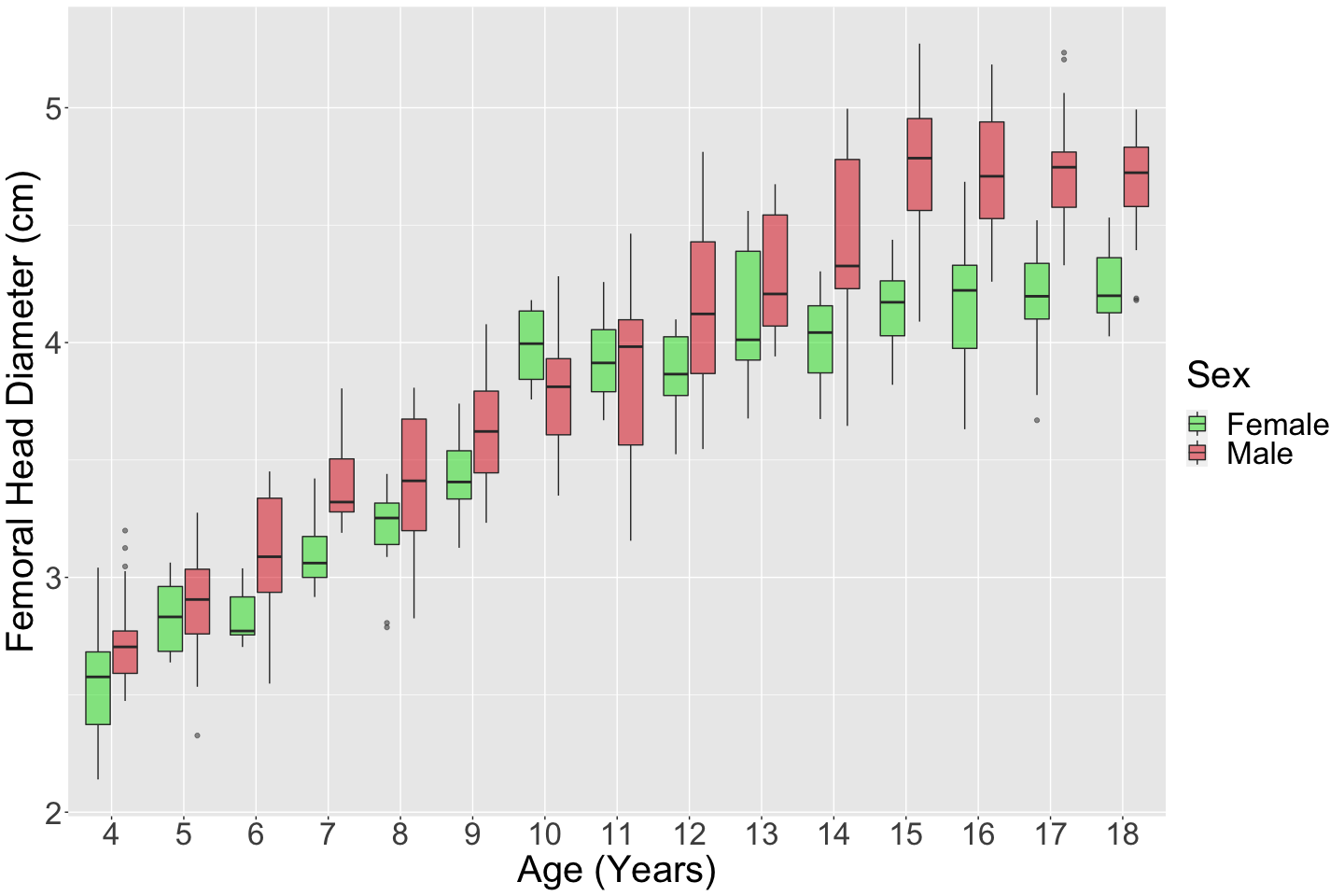  **E** \| 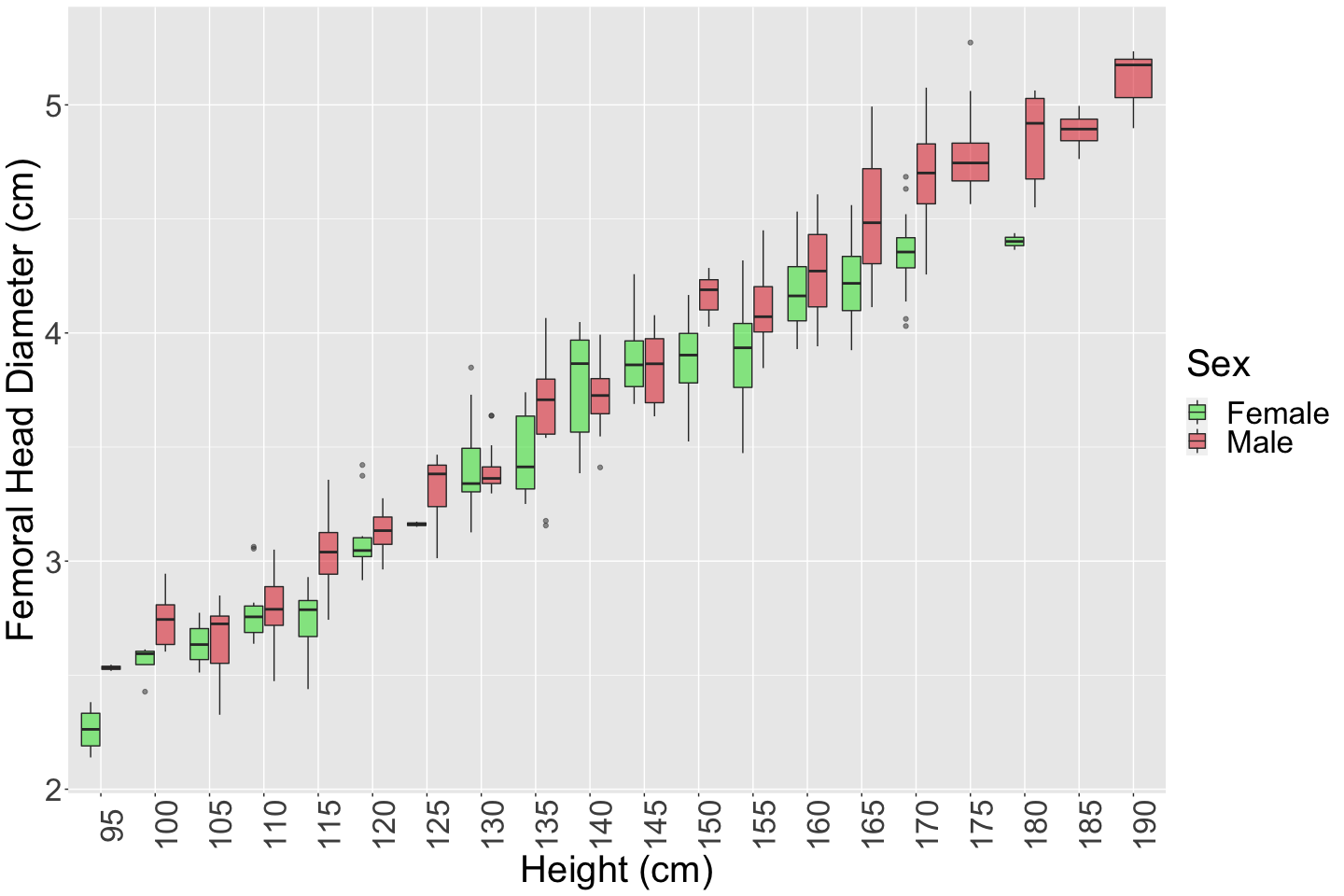  **F** \| \| **Femoral Length** \| 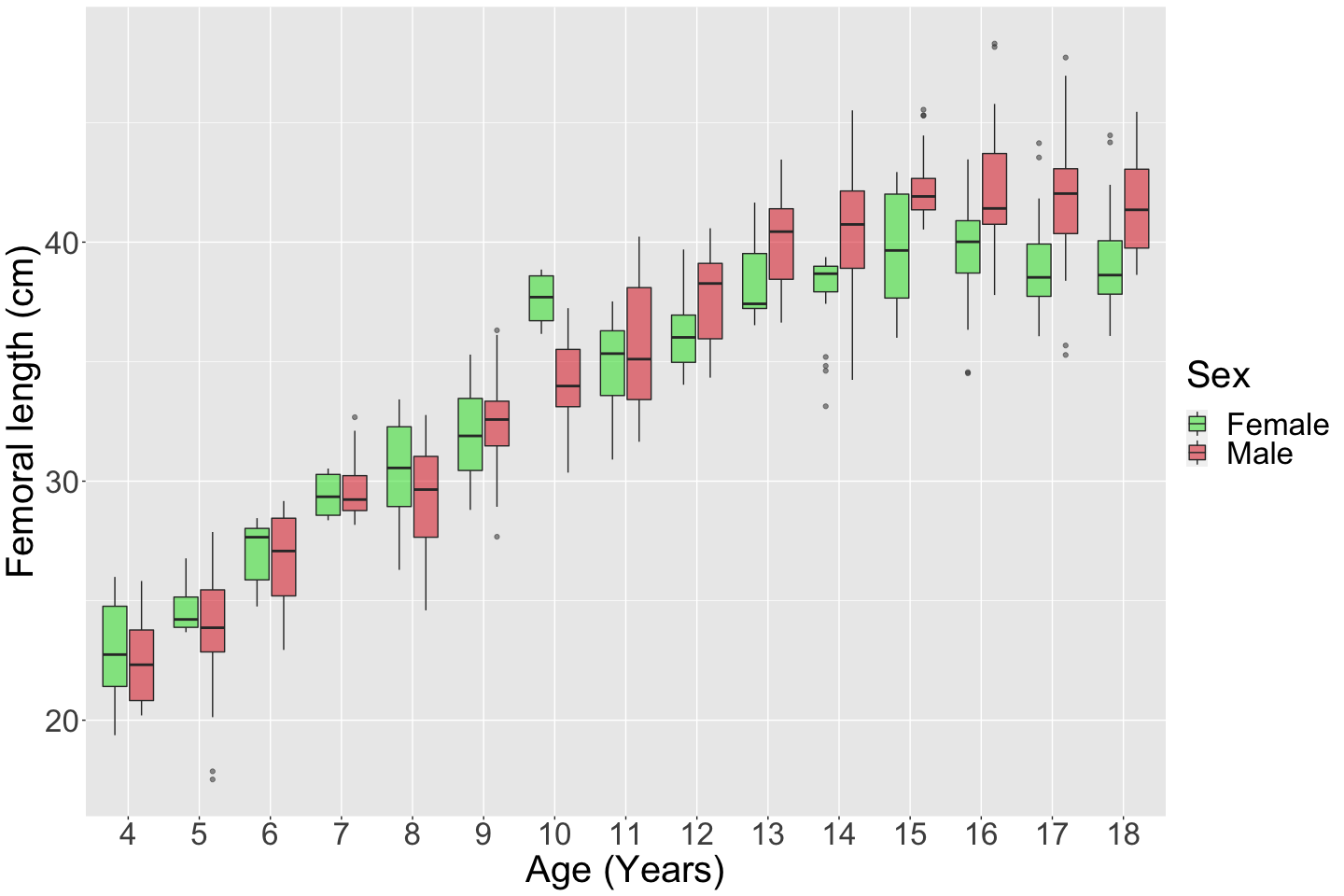  **G** \| 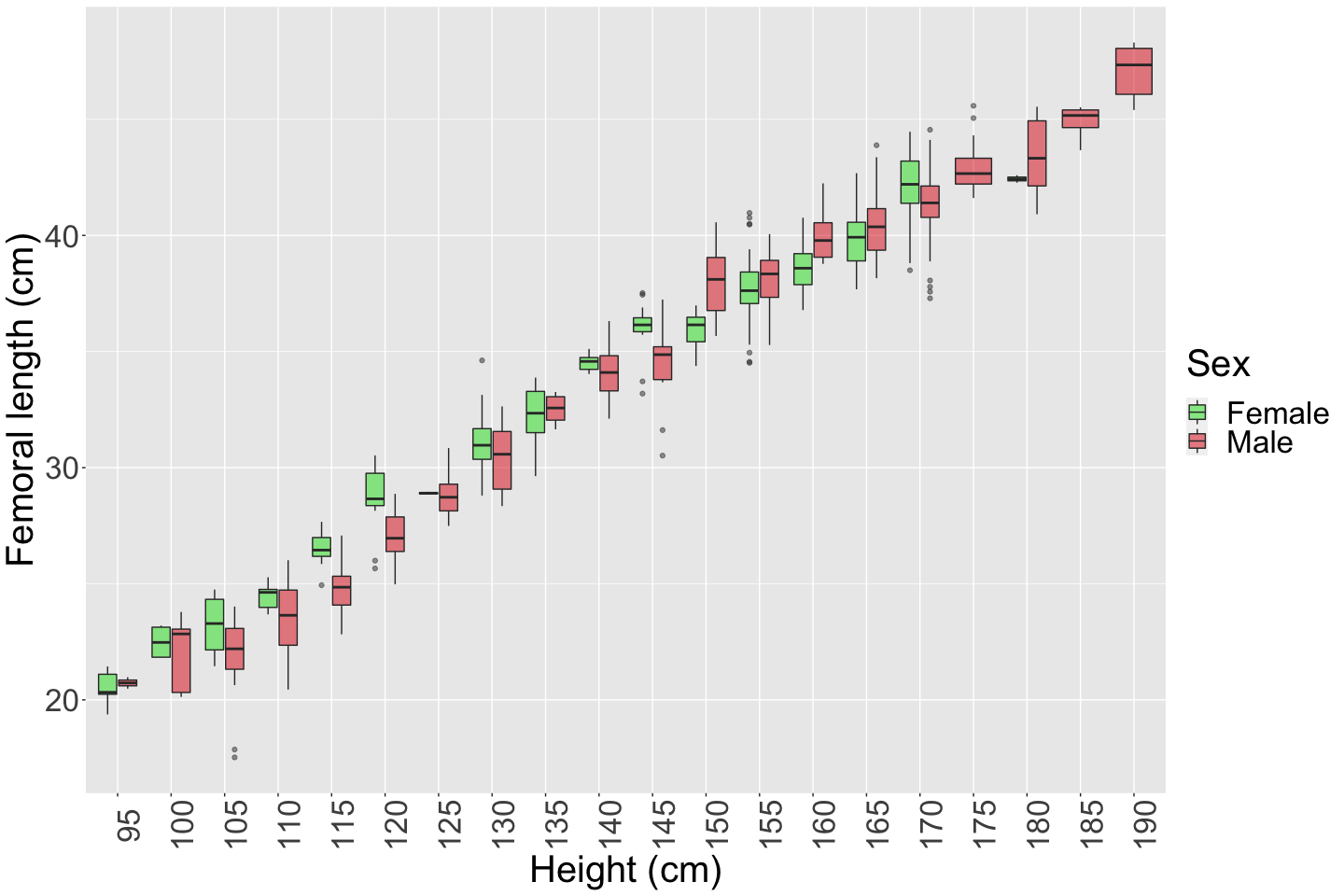  **H** \| |
| --- | --- | --- | --- | --- | --- | --- | --- | --- | --- | --- | --- | --- | --- | --- | --- | --- | --- | --- |

Figure S1: Femur angular and linear measurements; bicondylar angle (A&B), mLDFA (C&D) femoral head diameter (E&F) and femoral length (G&H) shown against age (A, C, E, and G) and height (B, D, F, and H)

| \|  \| **Tibia/Fibula** \| \| \| --- \| --- \| --- \| \|  \| **Age** \| **Height** \| \| **mMPTA** \| 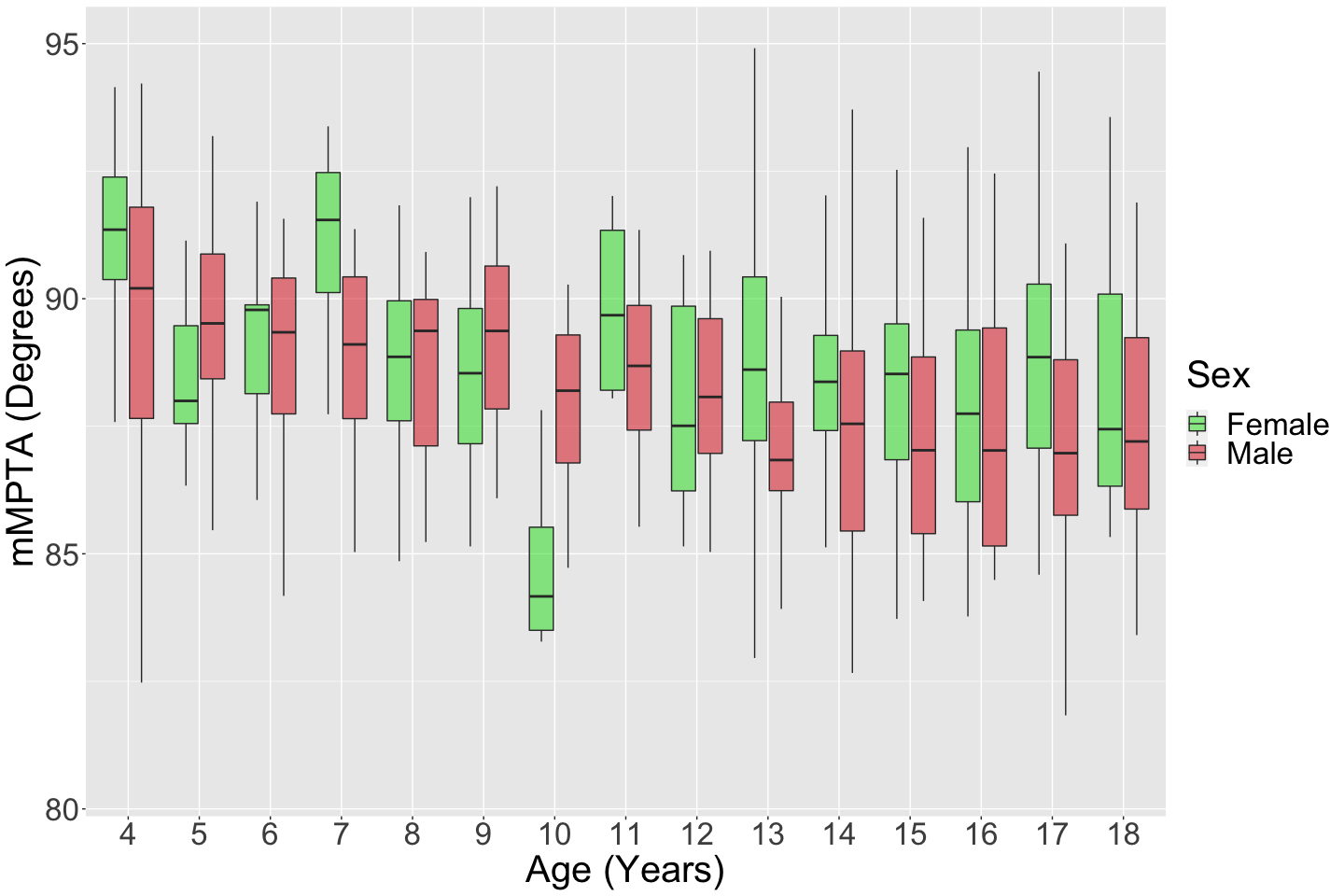  **A** \| 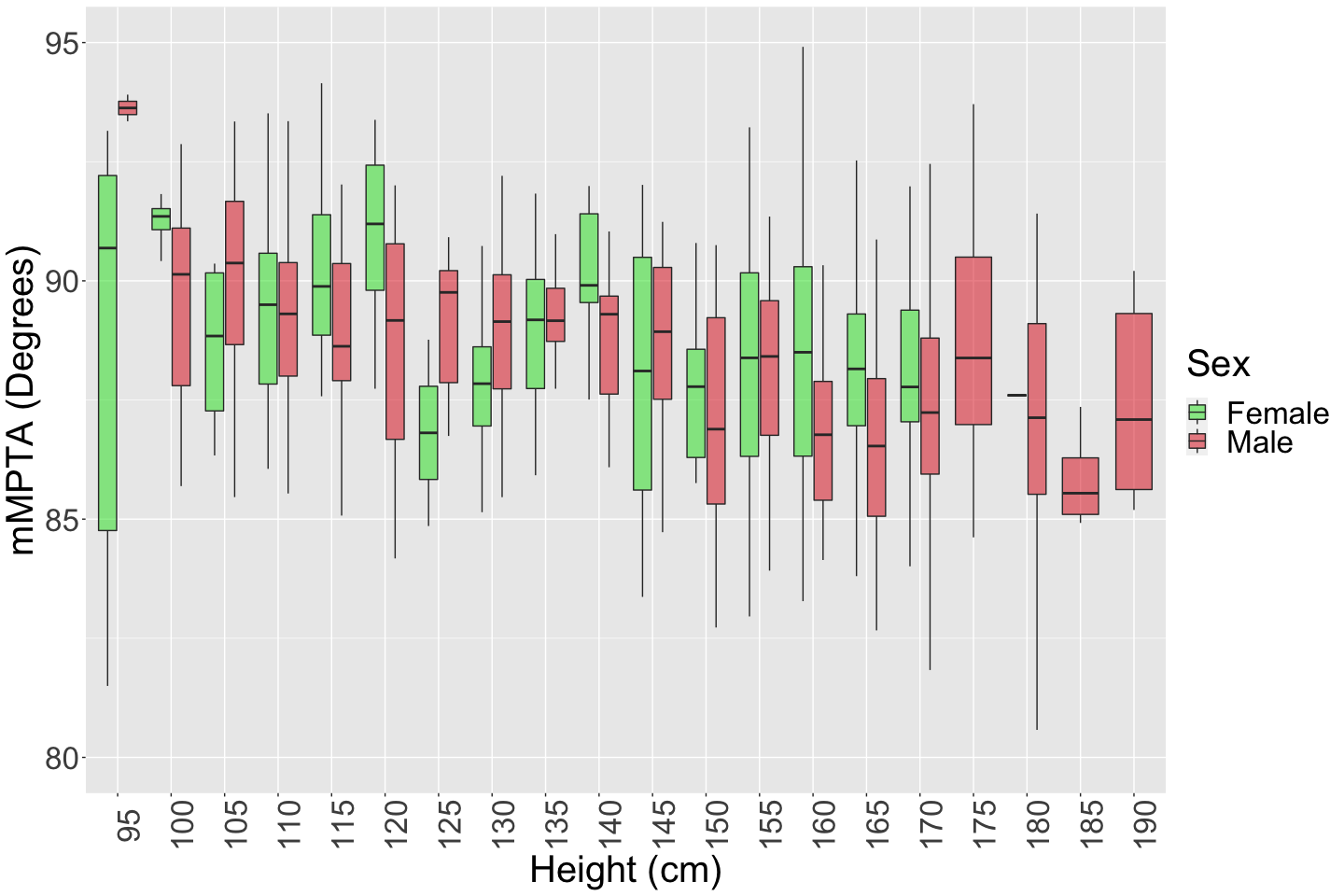  **B** \| \| **Condylar Width** \| 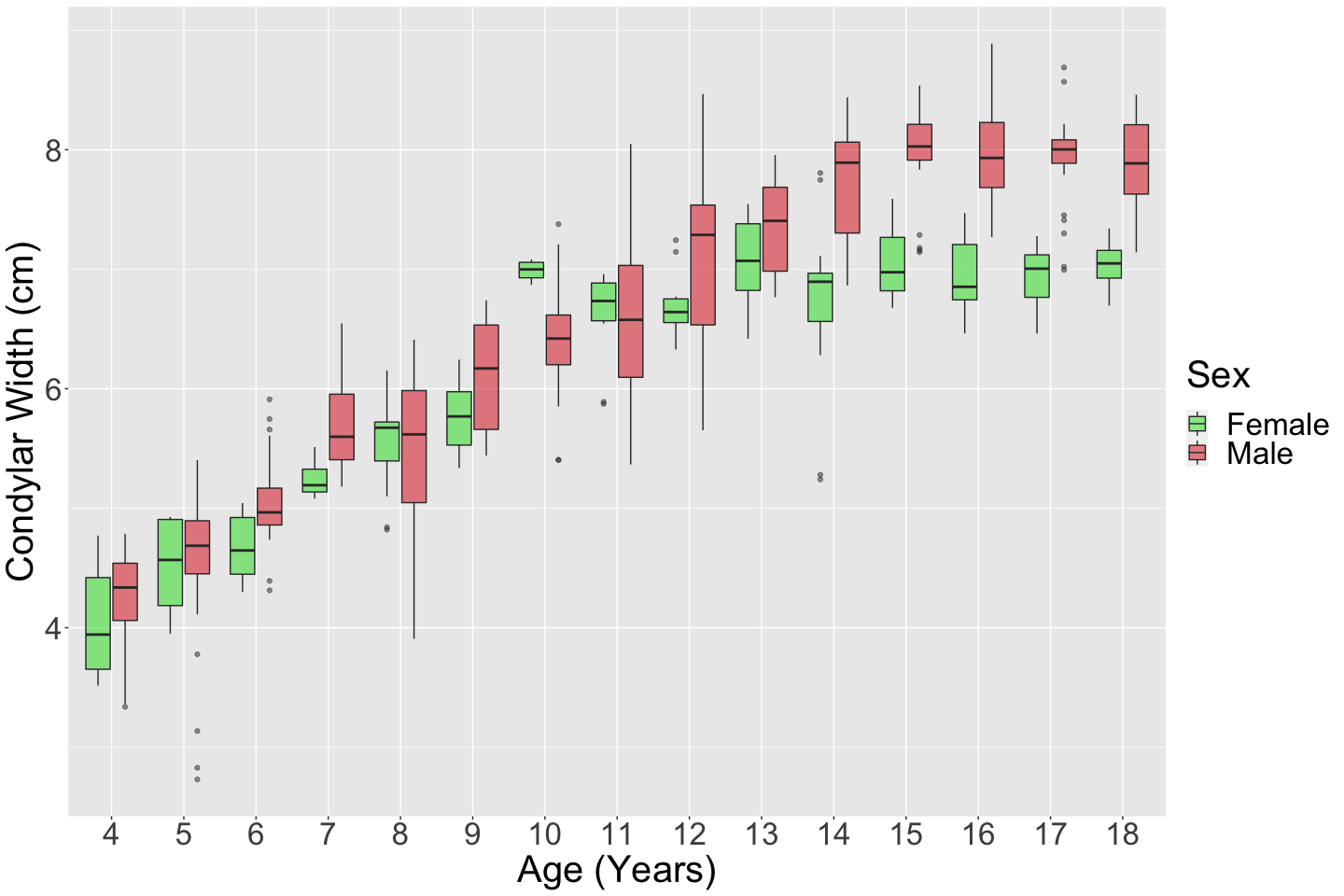  **C** \| 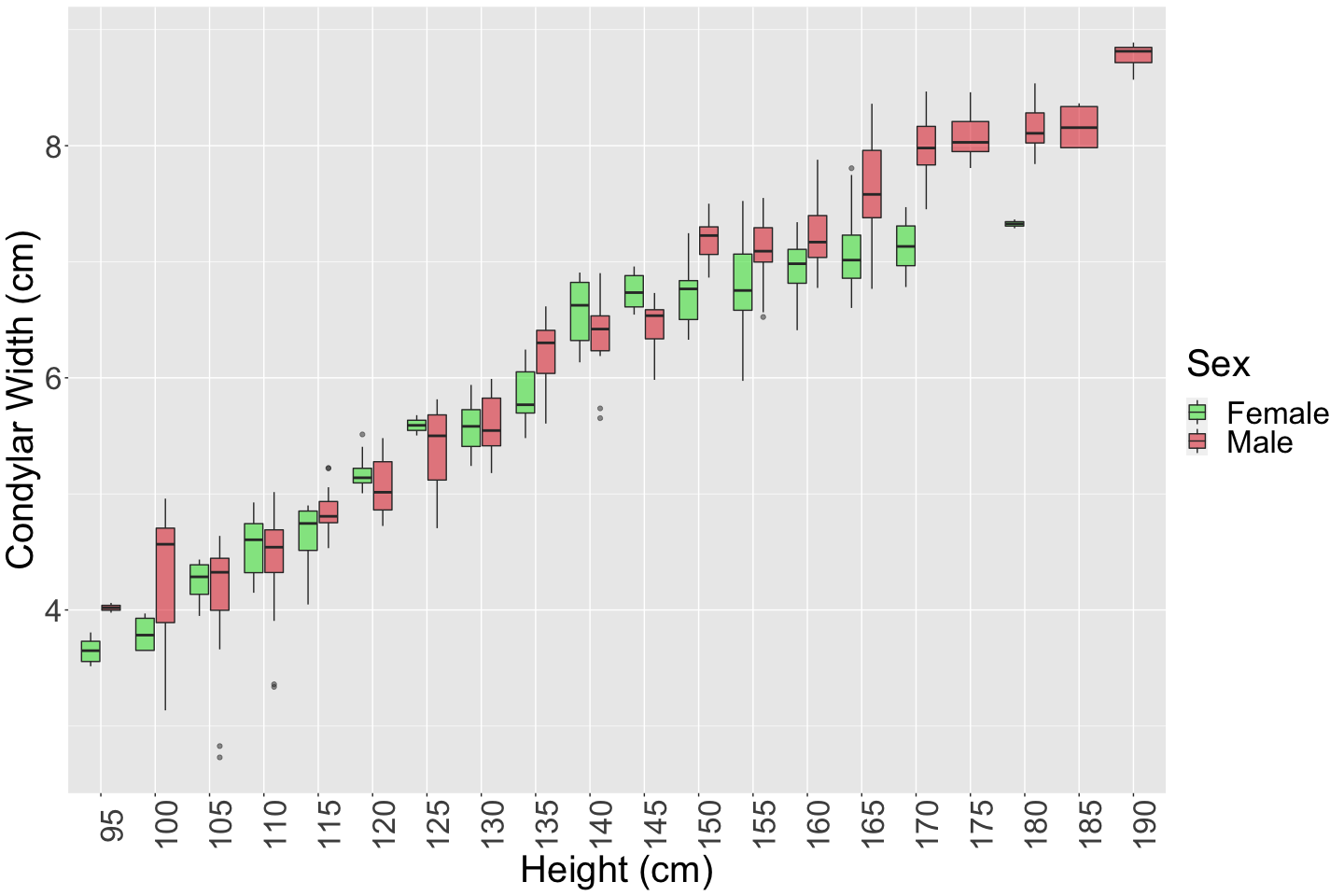  **D** \| \| **Tibial Length** \| 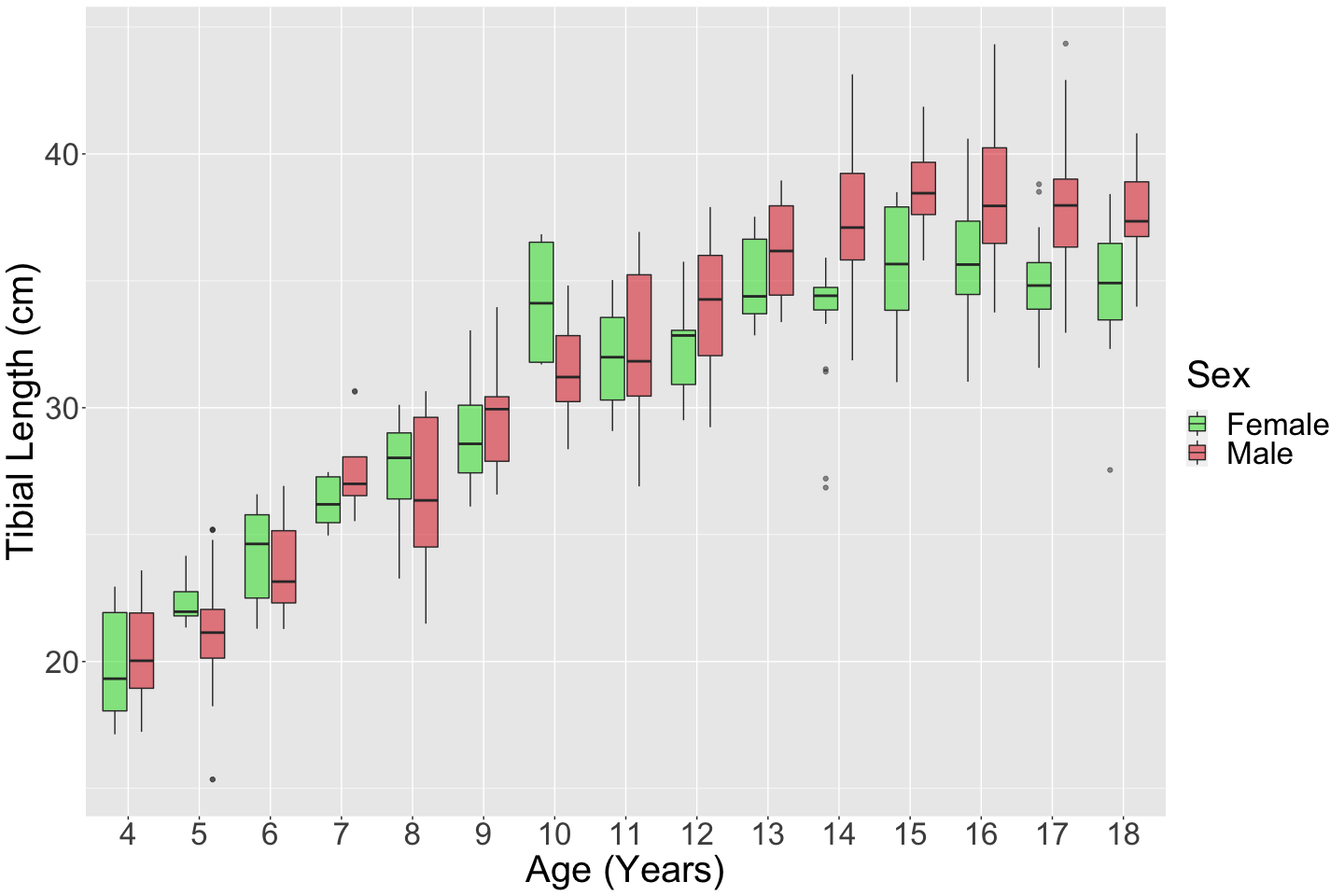  **E** \| 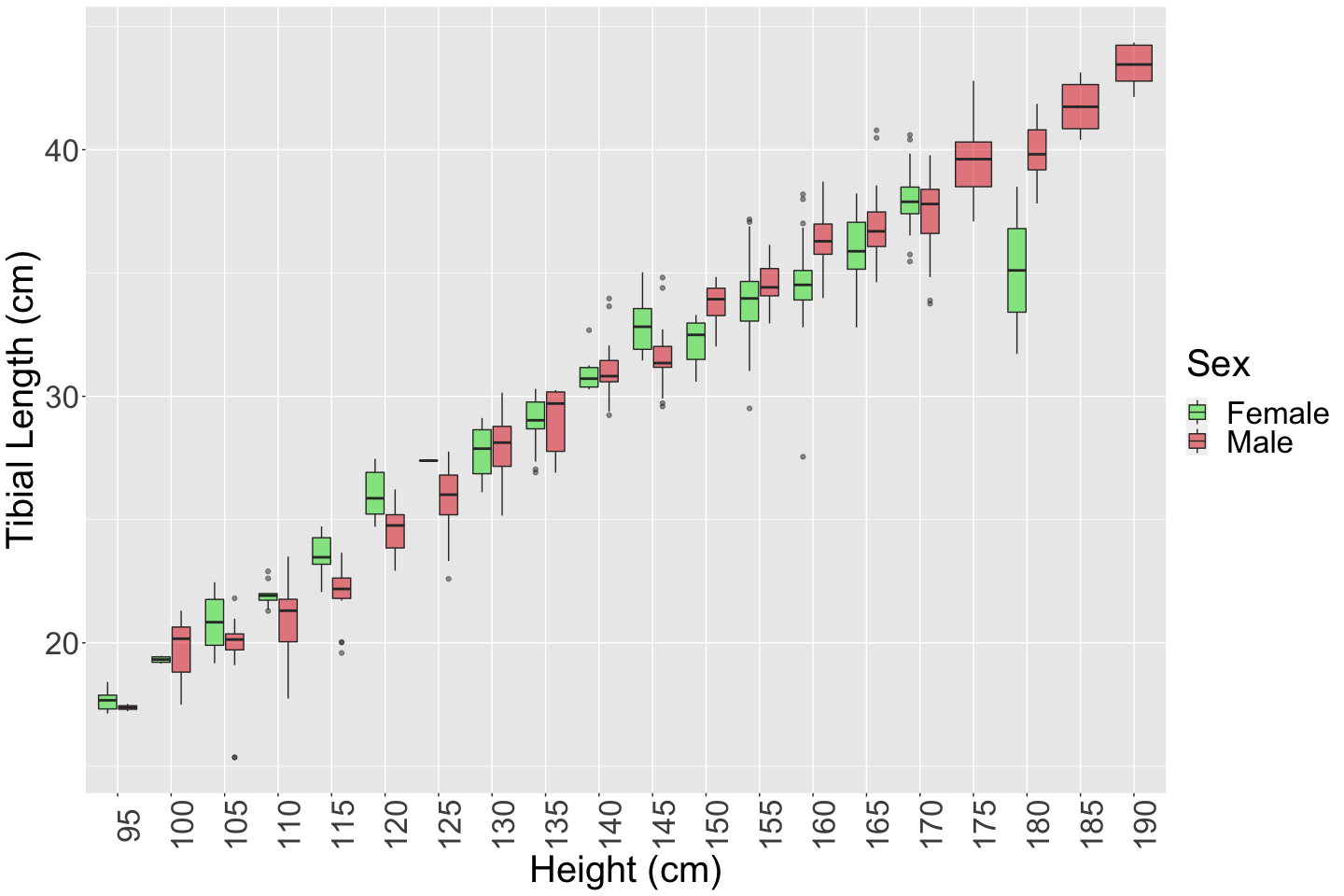  **F** \| |
| --- | --- | --- | --- | --- | --- | --- | --- | --- | --- | --- | --- | --- | --- | --- | --- |

Figure S2: Tibia/Fibula angular and linear measurements; mMPTA (A&B) condylar width (C&D) and tibial length (E&F) shown against age (A, C, and E) and height (B, D, and F)

| **Pelvis** | | |
| --- | --- | --- |
|  | **Age** | **Height** |
| **ASIS width** | 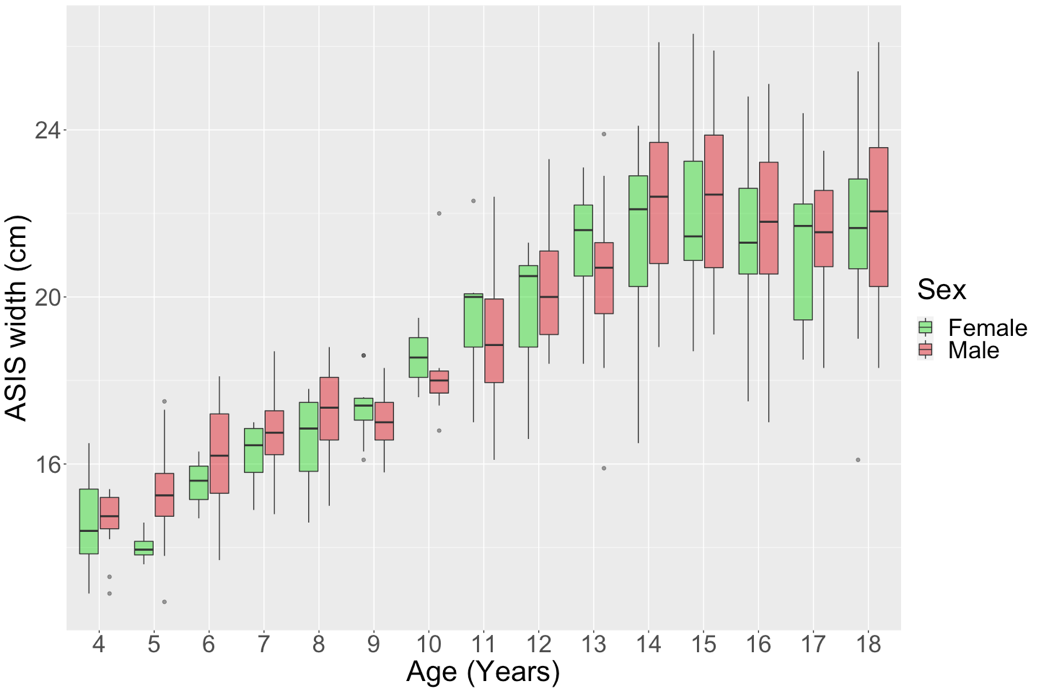  **A** | 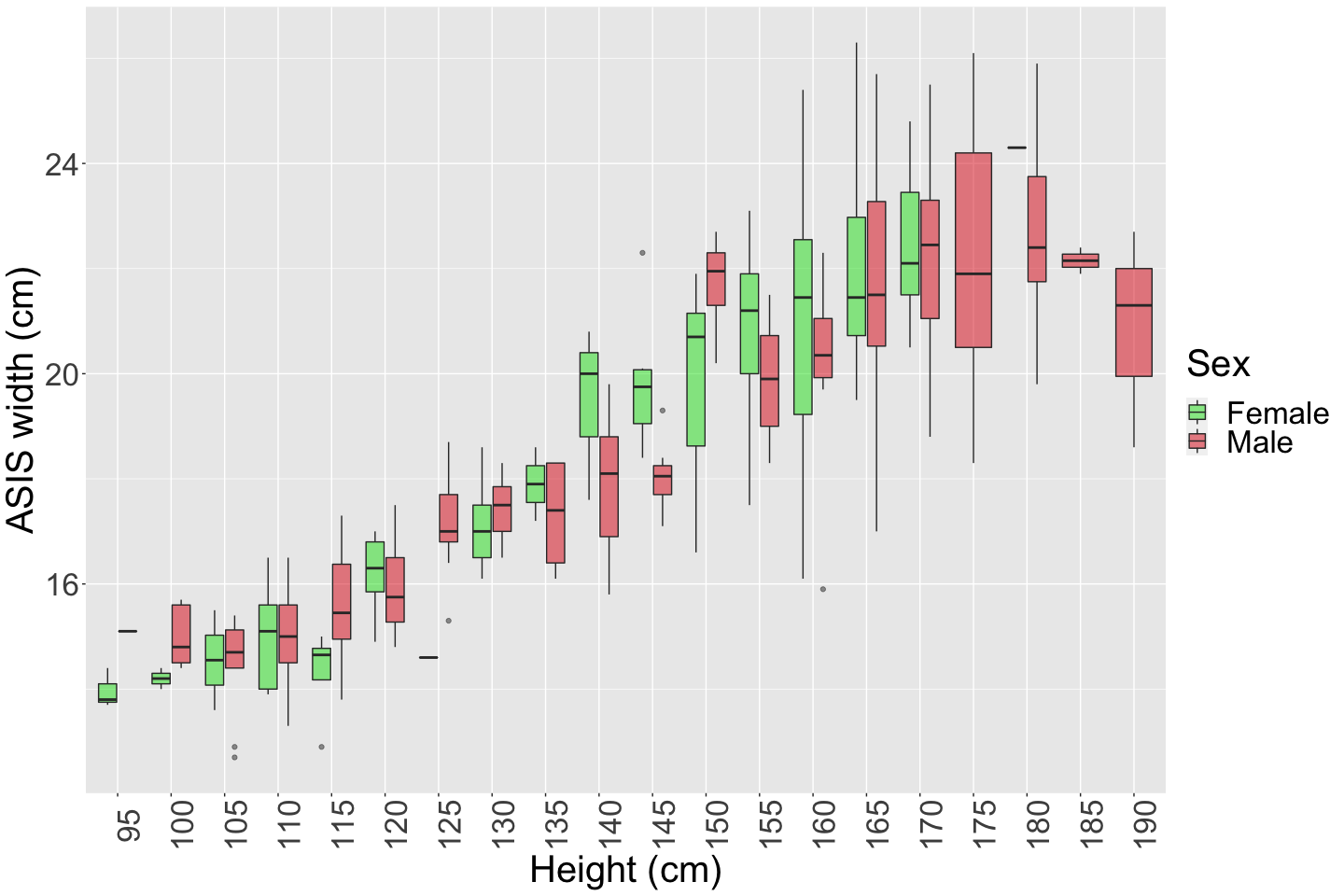  **B** |
| **Pelvis Depth** | 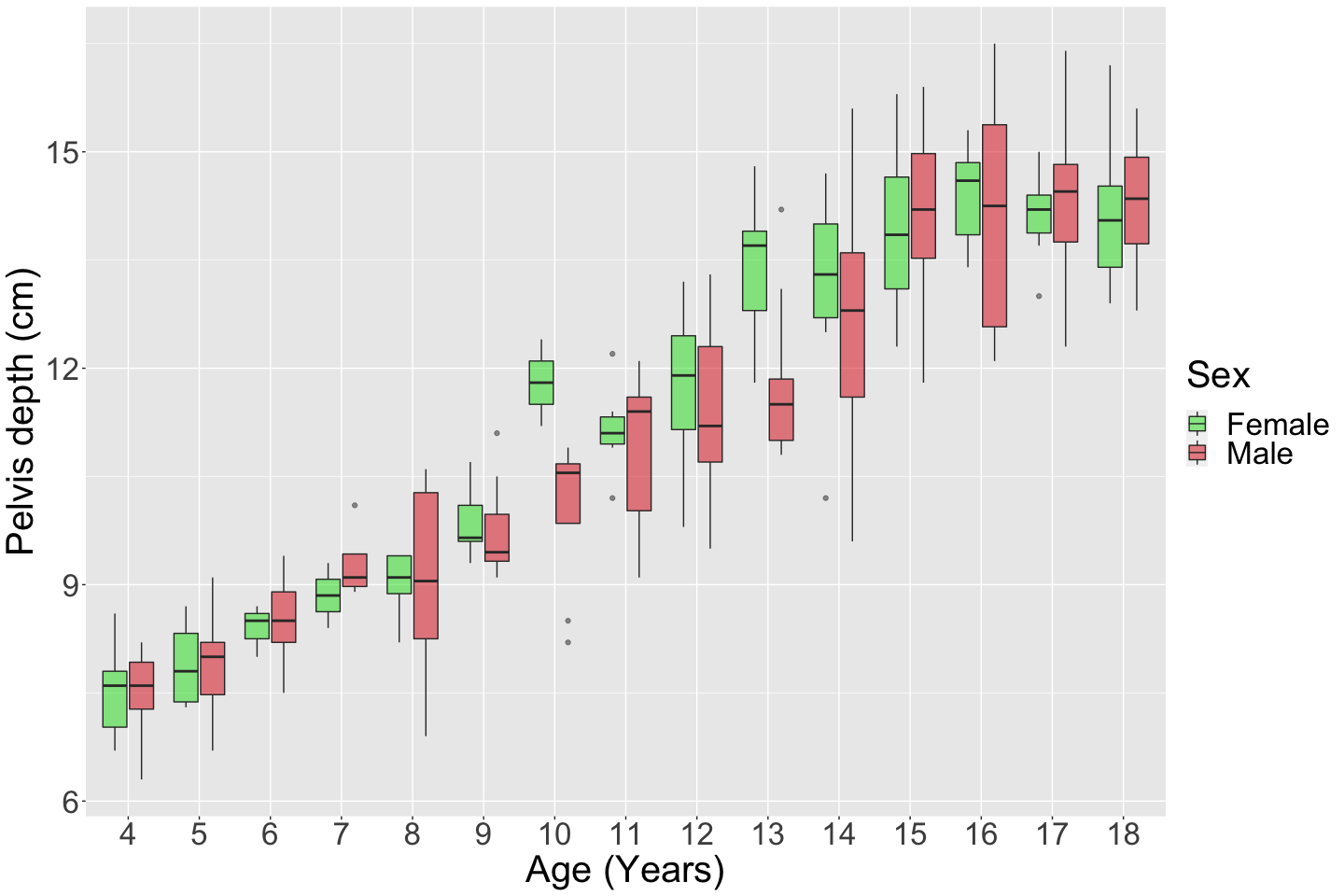  **C** | 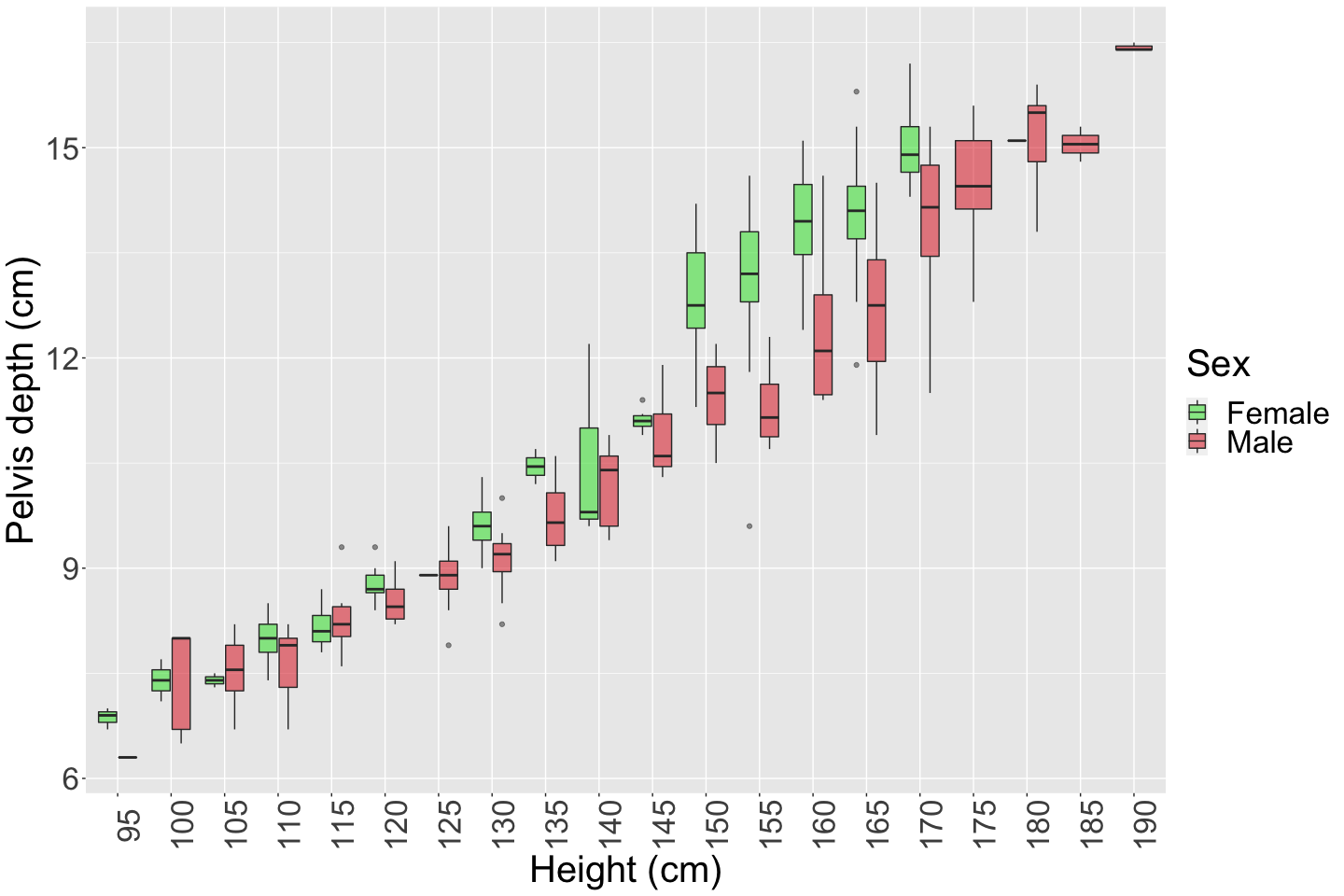  **D** |
| **Hip Joint Diameter** | 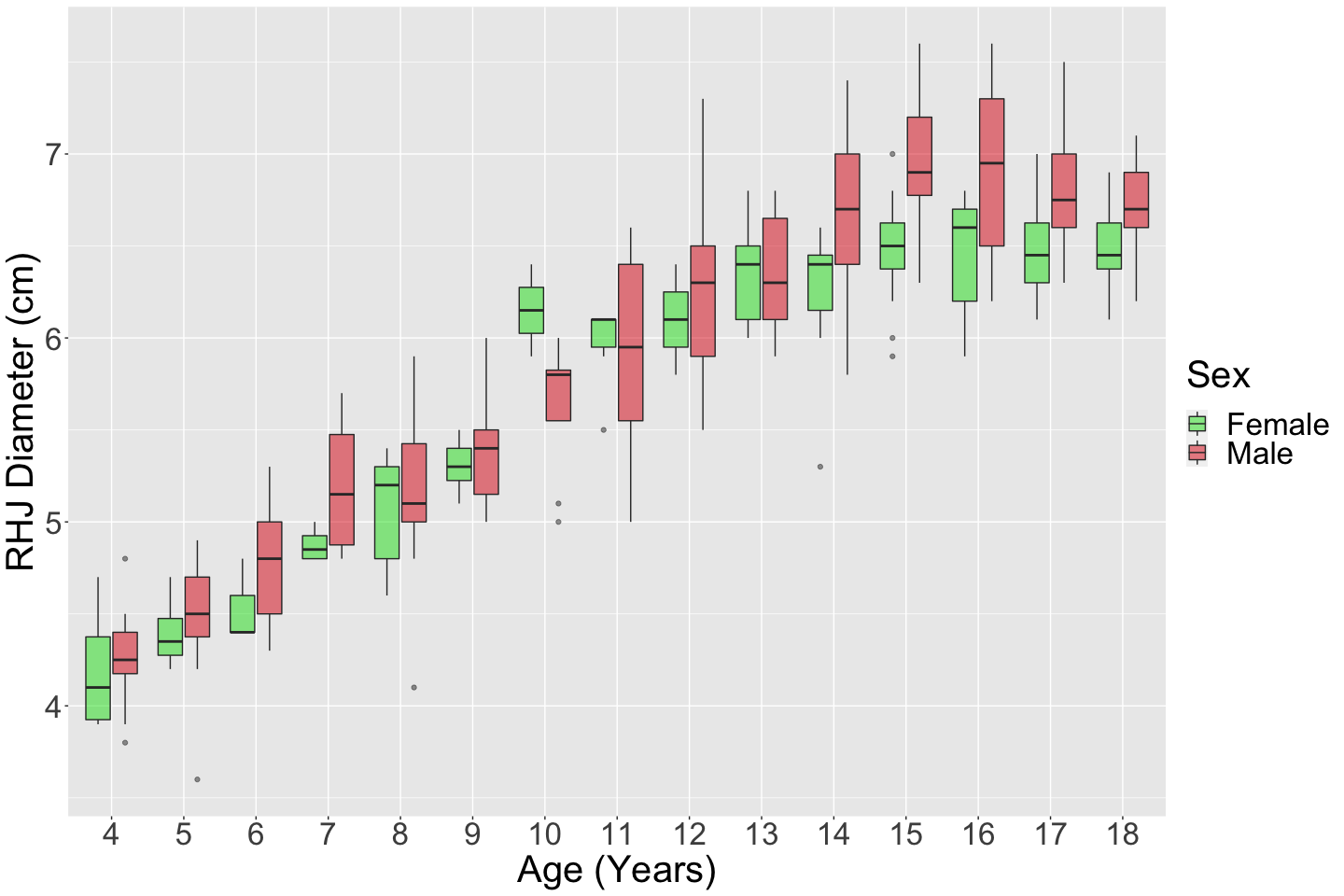  **E** | 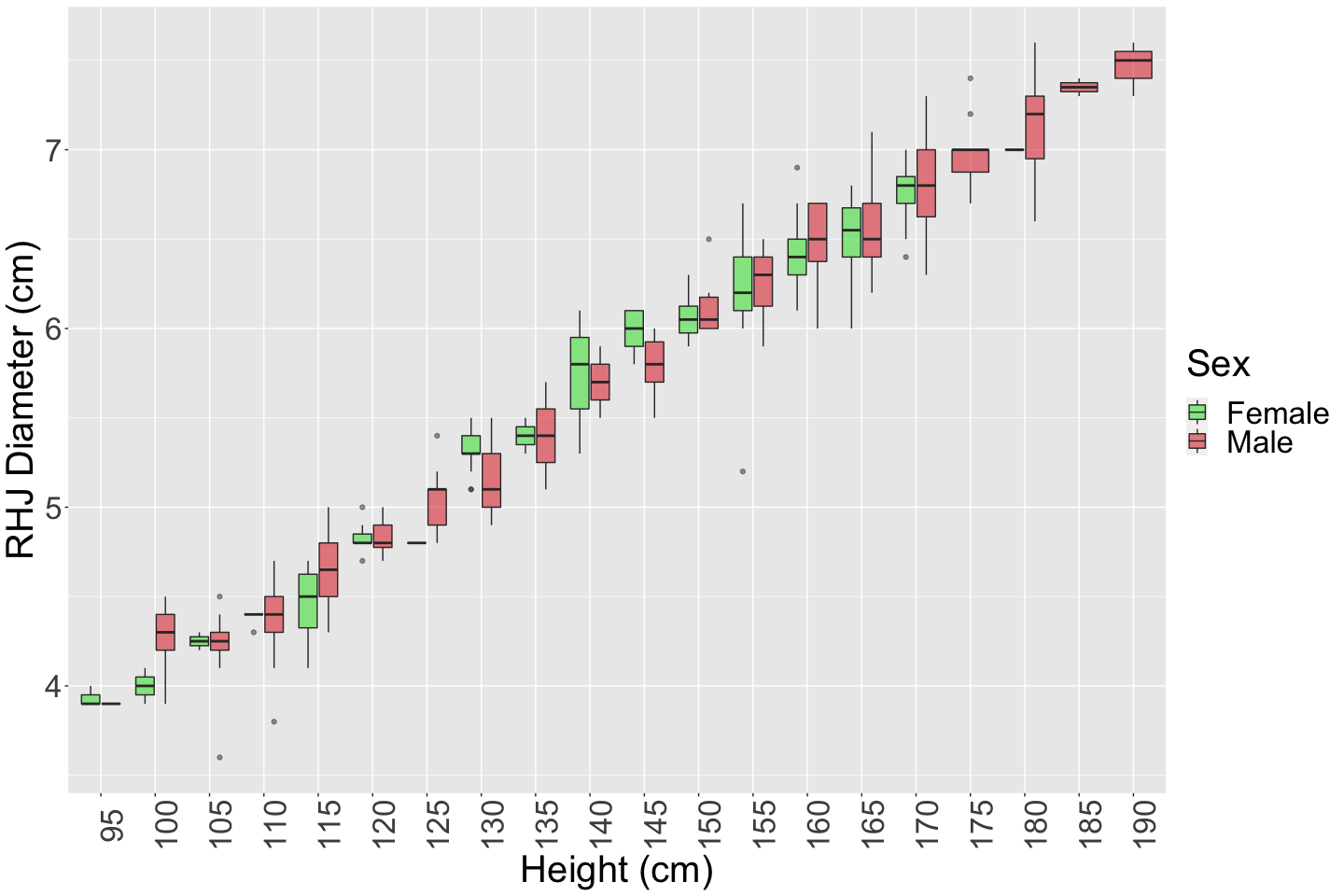  **F** |
| **Hip Joint Centre Distance** | 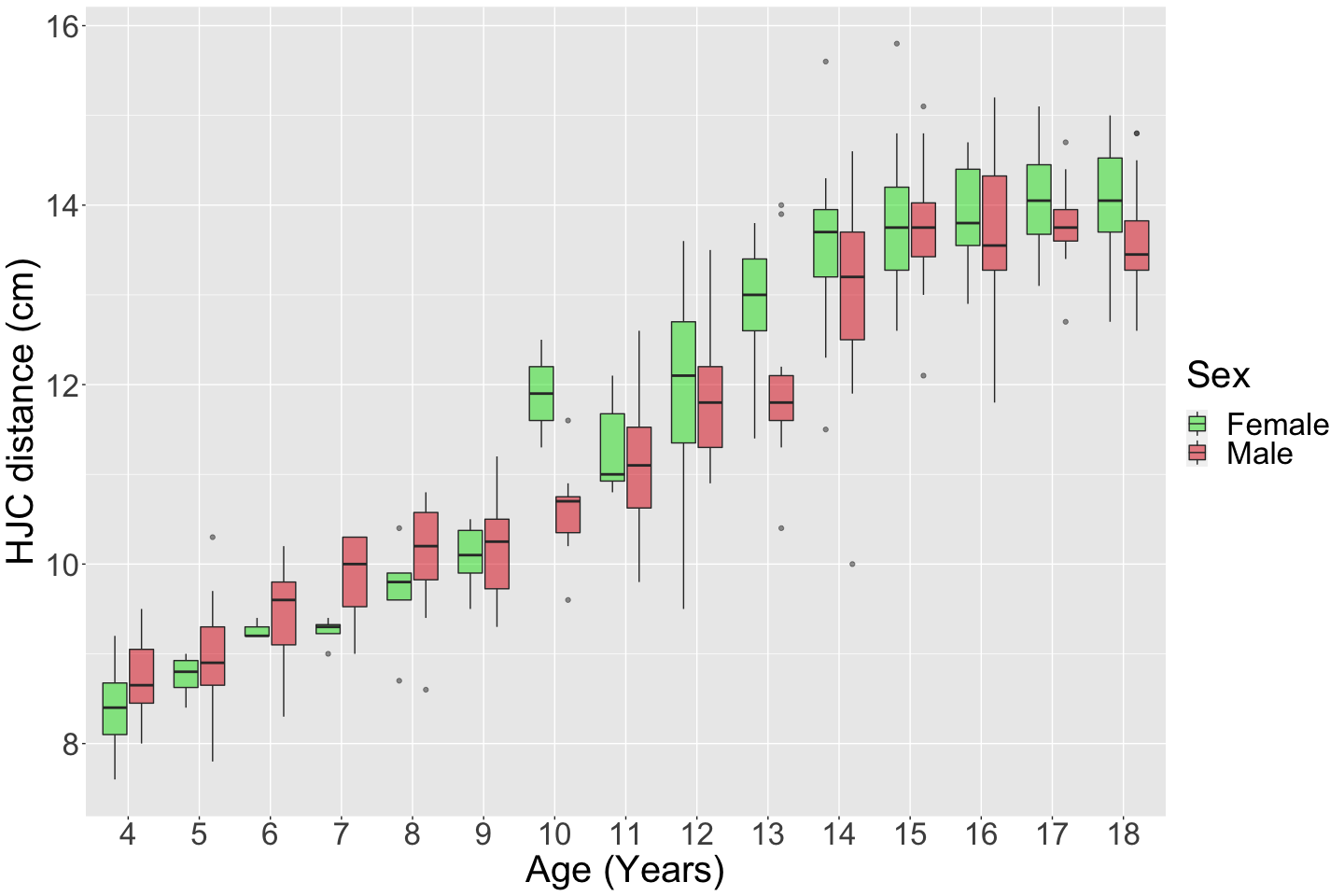  **G** | 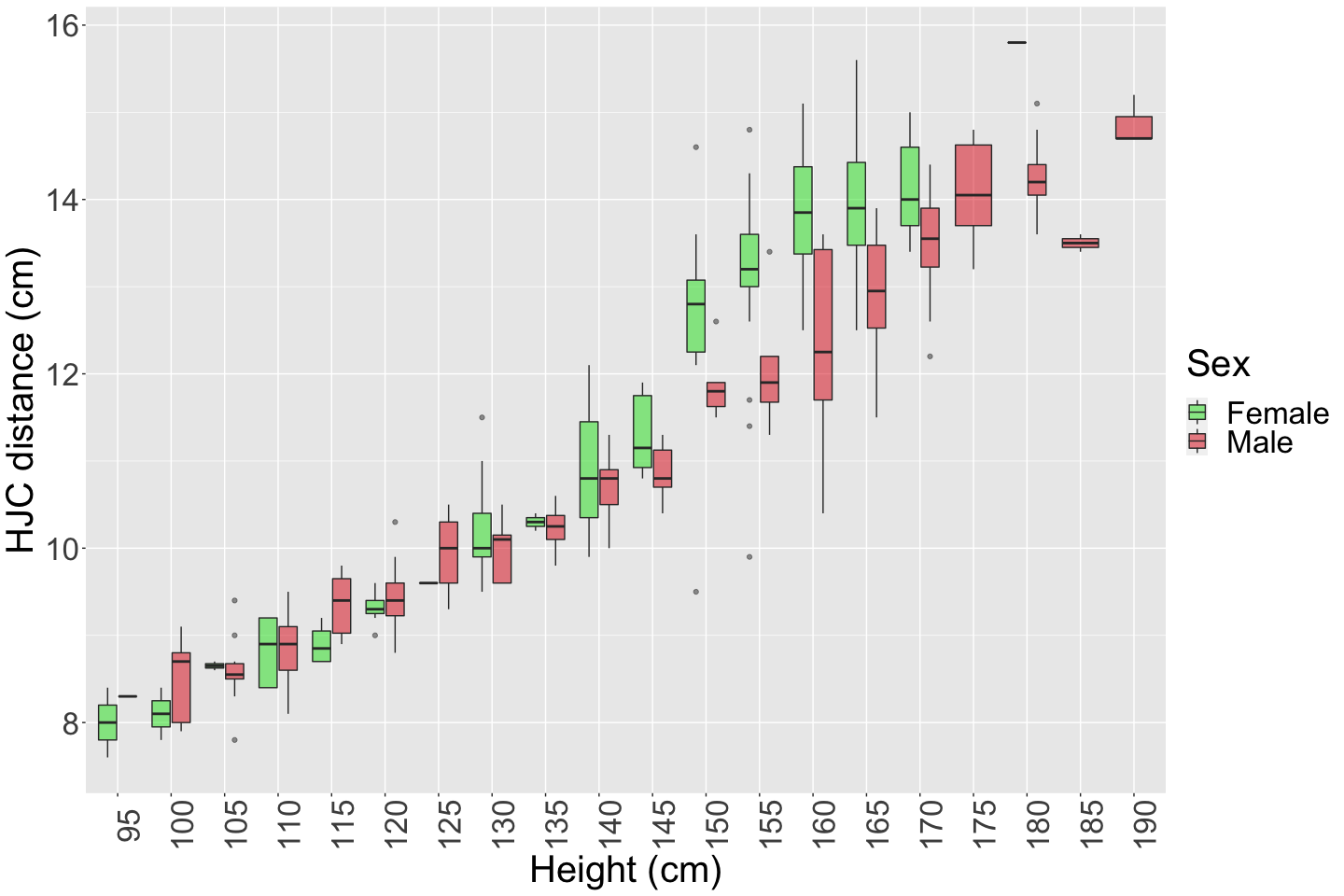  **H** |

|  |
| --- |

Figure S3: Pelvis linear measurements; ASIS width (A & B), pelvis depth (C&D), hip joint diameter (E&F) and hip joint centre distances (G&H) shown against age (A, C, E and G) and height (B, D, F and H).


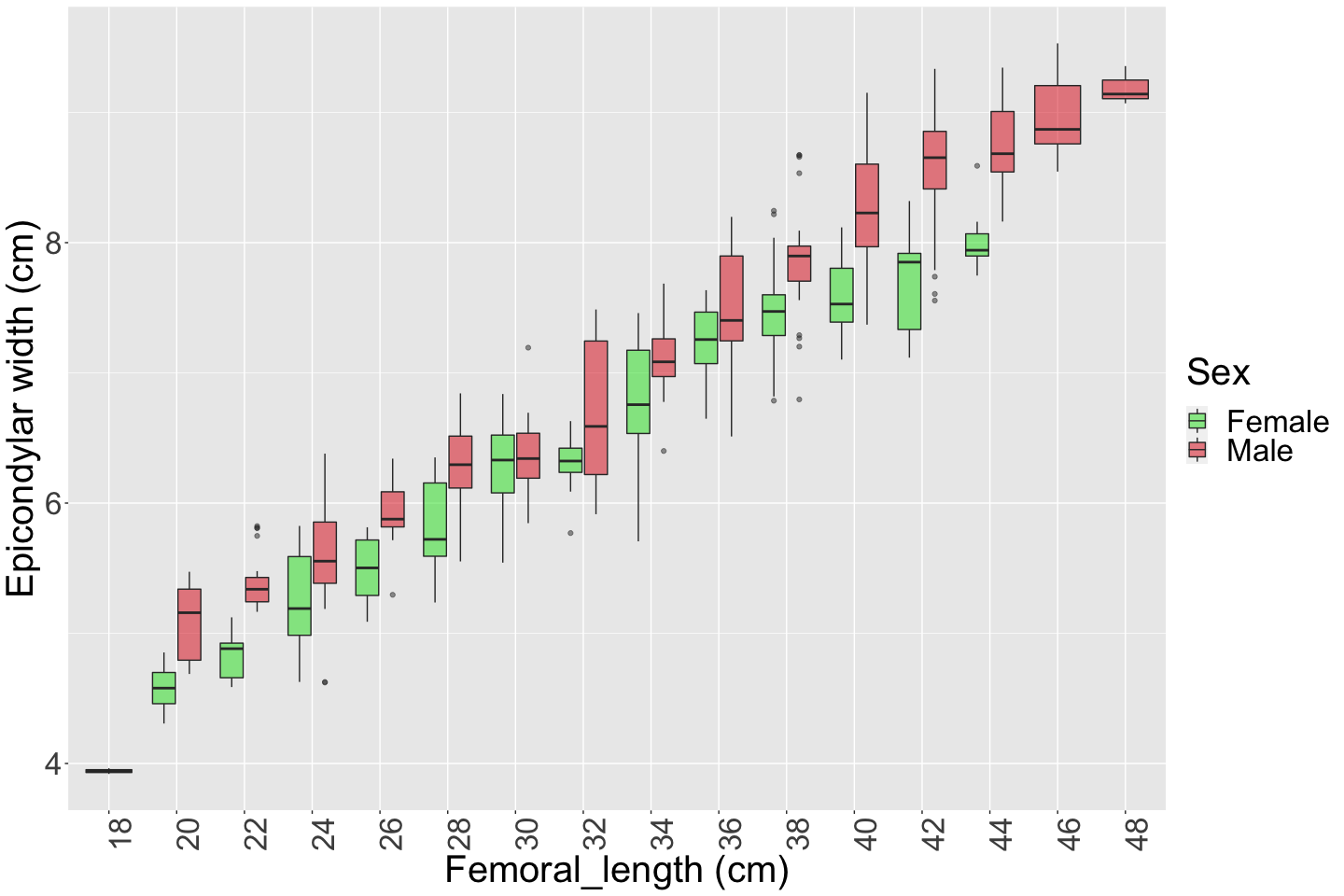


Figure S4: Epicondylar width vs. femoral length for children aged 4-18 years grouped by sex. Significant sex differences between males and females were observed between femoral lengths 28-29cm, and 38-45cm.


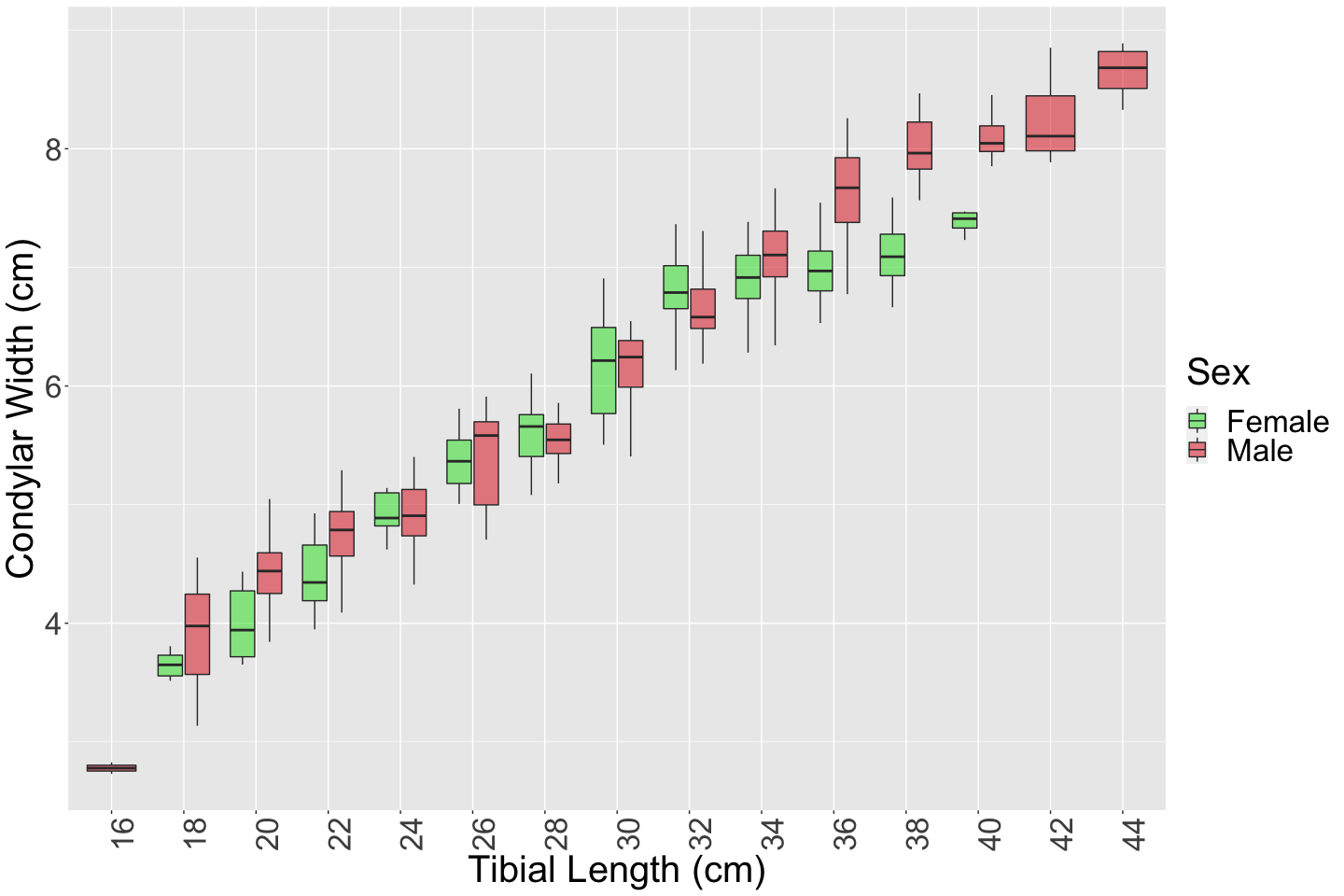

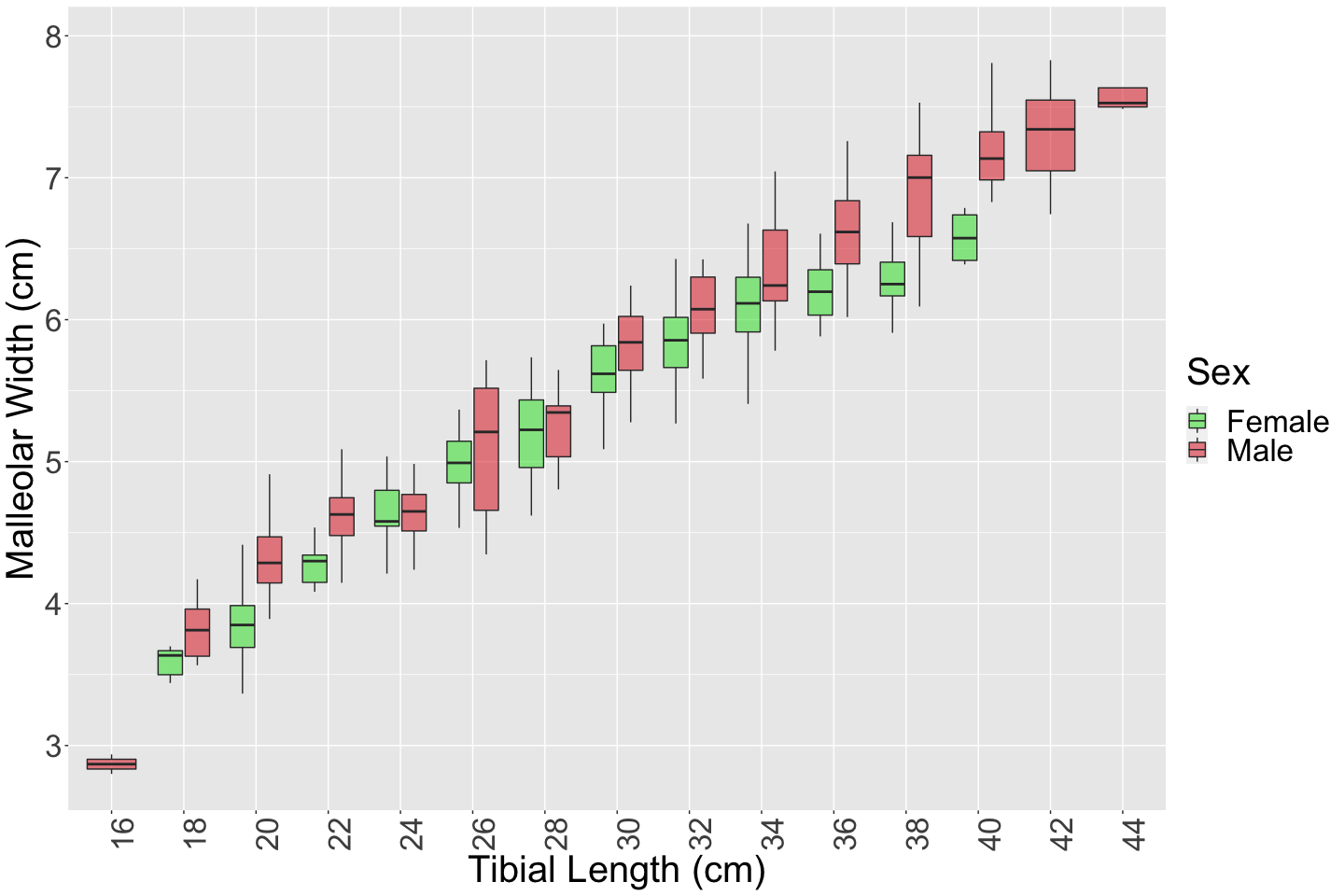


**B**

**A**

Figure S5: Condylar width A) and malleolar width (B) versus. tibial length measurements. Significant sex differences were observed between tibial lengths 36-39cm for both condylar and malleolar width


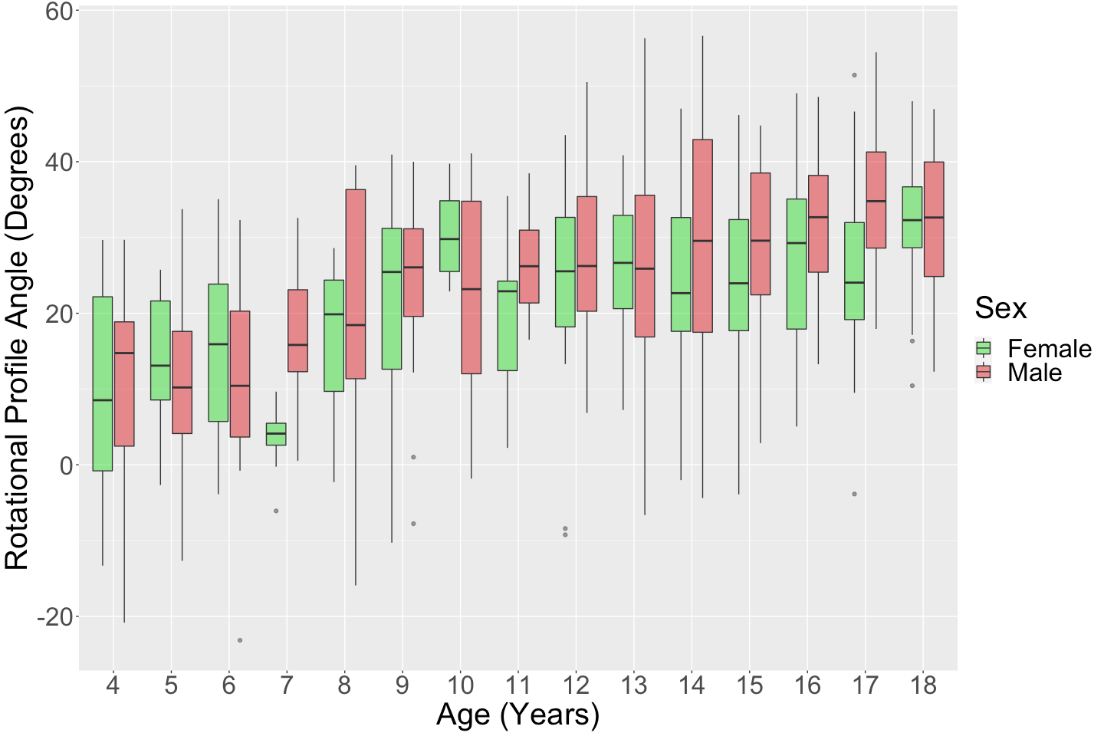

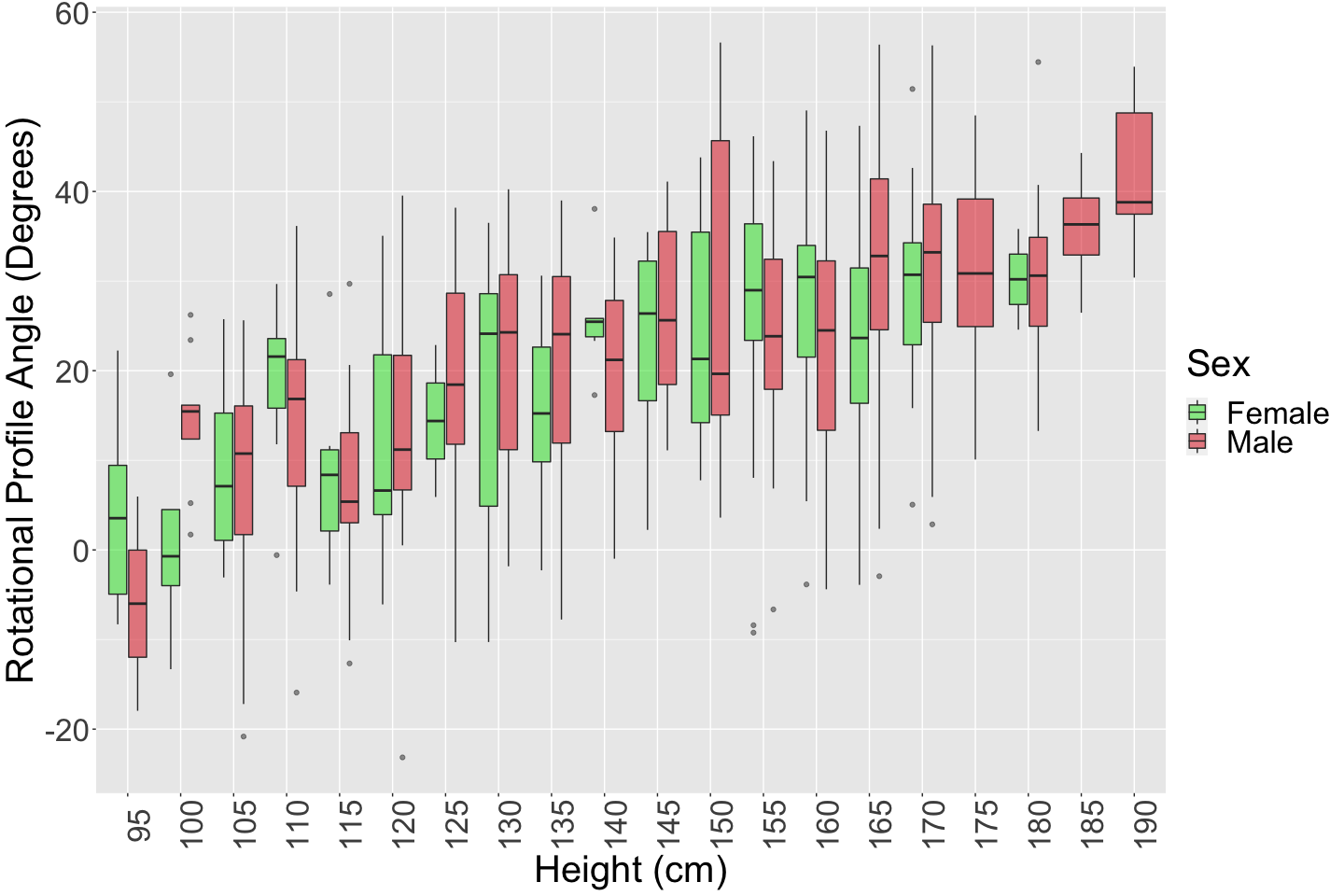


Figure S6: Rotational profile angle (tibial torsion - anteversion angle) vs. age (left) and height (right) for children aged 4-18 years grouped by sex
